# Nanoengineering of Phosphate/Phosphonate Drugs via Competitive Replacement with Metal‐Phenolic Networks to Overcome Breast Tumor with Lung and Bone Metastasis

**DOI:** 10.1002/advs.202413201

**Published:** 2024-11-18

**Authors:** Wanrui Shi, Dashuai Liu, Wenjie Feng, Yang Chen, Yonggang Wang, Zhihong Nie, Yi Liu, Hao Zhang

**Affiliations:** ^1^ Joint Laboratory of Opto‐Functional Theranostics in Medicine and Chemistry Institute of Translational Medicine The First Hospital of Jilin University Changchun 130021 P. R. China; ^2^ State Key Laboratory of Supramolecular Structure and Materials College of Chemistry Jilin University Changchun 130012 P. R. China; ^3^ Department of Cardiovascular Centre The First Hospital of Jilin University Changchun 130012 P. R. China; ^4^ State Key Laboratory of Molecular Engineering of Polymers Department of Macromolecular Science Fudan University Shanghai 200438 P. R. China

**Keywords:** competitive coordination, metal‐phenolic networks, nanoengineering, phosphates and phosphonates, self‐assembly

## Abstract

Phosphate and phosphonate drugs are vital in building organisms, regulating physiological processes, and exhibiting diverse biological activities, including antiviral, antibacterial, antineoplastic, and enzyme‐inhibitory effects. However, their therapeutic potential is limited by the lack of advanced nanoengineering technologies. Herein, a competitive coordination strategy for nanoengineering phosphate/phosphonate drugs is introduced. By leveraging the difference in coordination capabilities between polyphenols and phosphates/phosphonates with metal ions, various phosphate/phosphonate‐based nanodrugs using metal‐phenolic networks (MPNs) as templates and phosphate/phosphonate drugs as competitive agents are constructed. The dynamic nature of these coordination bonds imparts stimuli‐responsiveness to the nanodrugs, allowing for targeted release and therapy. As a proof of concept, Fe^3+^ and galangin are used to form the MPN template, zoledronic acid and cGAMP as competitive agents, and DOX as the loaded drug to construct DOX@Fe‐galangin@Fe‐zoledronic acid‐cGAMP nanodrugs. The results demonstrate that, by triggering pyroptosis and activating the cGAS‐STING pathway, the nanodrugs exhibit potent cytotoxicity and accurate selectivity in eradicating orthotopic breast tumors, and activate an antitumor immune response against lung and bone metastases. Because the competitive coordination strategy is applicable to a variety of phosphate/phosphonate agents, it holds significant potential for enhancing the clinical efficacy of phosphate/phosphonate drugs and advancing nanodrug development for complex therapeutic applications.

## Graphical abstract

A competitive coordination strategy is introduced for nanoengineering phosphate/phosphonate drugs by leveraging differences in coordination capabilities between polyphenols and phosphates/phosphonates with metal ions. This approach allows for the construction of various stimuli‐responsive phosphate/phosphonate‐based nanodrugs, using metal‐phenolic networks as templates and phosphate/phosphonate drugs as competitive agents.

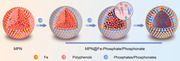

## 1 Introduction

Natural phosphates and phosphonates are one class of the most essential compounds in the human body, serving as fundamental components (nucleotides, phospholipids, etc.) for constructing living organisms^[^ ^1^ ^]^ and as signaling molecules (adenosine triphosphate (ATP), cyclic dimeric adenosine monophosphate (CDA), cyclic adenosine monophosphate (cAMP), etc.) for regulating physiological processes.^[^ ^2^ ^]^ Simultaneously, synthetic phosphates/phosphonates have demonstrated a wide range of biological activities like antiviral, antibacterial, antineoplastic, and enzyme‐inhibitory effects. For instance, monophosphates/monophosphonates such as tenofovir and cidofovir are potent therapeutics against cancer and viral infections caused by human immunodeficiency virus (HIV), hepatitis B and C viruses (HBV and HCV), and SARS‐CoV‐2.^[^ ^3^ ^]^ Bisphosphonates, including zoledronic acid, minodronate, risedronate, and alendronate, are clinically effective in treating bone‐resorption disorders, such as osteoporosis, Paget's disease, metastatic bone disease, and myeloma bone disease by inhibiting farnesyl pyrophosphate synthase.^[^ ^4^ ^]^ Phosphate derivatives of glucocorticoids, such as prednisolone disodium phosphate, betamethasone 21‐phosphate sodium, and dexamethasone 21‐phosphate disodium salt play a vital role in down‐regulating the expression of the interleukin six gene and other cytokine genes, thereby reducing inflammatory and immune responses.^[^ ^5^ ^]^ Furthermore, many nucleoside analogs, including 5‐fluorouracil and gemcitabine, exert their therapeutic effects after being converted in vivo into their mono‐, di‐, and triphosphates.^[^ ^6^ ^]^ Due to structural differences from natural nucleosides, the efficiency of their phosphorylation by nucleoside/nucleotide kinases to generate active metabolites is often limited, with the first phosphorylation being the rate‐limiting step. Thus, nucleoside monophosphate analogs are developed to bypass this initial phosphorylation step, significantly enhancing their therapeutic efficacy.^[^ ^6^, ^7^ ^]^ Despite the fascinating biological activities, the therapeutic potential of phosphates/phosphonates is considerably limited by the inherent challenges of small molecule drugs, including rapid renal clearance,^[^ ^8^ ^]^ brief half‐life,^[^ ^9^ ^]^ restricted cellular permeability,^[^ ^10^ ^]^ and severe side effects.^[^ ^11^ ^]^ Nevertheless, advancements in drug delivery nanotechnology offer an exceptional avenue to overcome these challenges.^[^ ^2^, ^12^ ^]^ The development of nano drug delivery systems promises to markedly enhance the solubility, stability, biocompatibility, and bioavailability of phosphates/phosphonates, opening new pathways for their improved clinical use.

To date, most phosphate/phosphonate‐based nanodrug delivery systems rely on nanocarriers, including polymeric (micelles, nanospheres, etc.)^[^ ^12^, ^13^ ^]^ and inorganic (mesoporous silica nanoparticles,^[^ ^14^ ^]^ hydroxyapatite nanocrystals,^[^ ^15^ ^]^ and metal–organic frameworks (MOFs),^[^ ^12^, ^16^ ^]^ etc.) variants. However, polymeric nanocarriers often suffer from diminished stability in the bloodstream, as well as potential cytotoxicity and hemolysis.^[^ ^17^ ^]^ Inorganic nanocarriers raise concerns regarding long‐term toxicity and stability due to their non‐biodegradable nature.^[^ ^18^ ^]^ Moreover, the majority of these nanocarriers are limited by their complex and costly manufacturing processes and relatively low drug‐loading capacity.^[^ ^19^ ^]^ The emerging field of metal‐phenolic networks (MPNs) presents a promising alternative. MPNs are coordination complexes formed between metal ions (Fe^3+^, Cu^2+^, Mn^2+^, and Gd^3+^, etc.) and phenolic ligands (phenolic acids, flavonoids, stilbenes, and lignans, etc.). These networks not only combine the diagnostic and therapeutic benefits of metal ions and phenolic ligands but also take advantage of the dynamic and stable nature of coordinated covalent bonds.^[^ ^20^ ^]^ The library of phenolic ligands (synthetic and naturally abundant phenolics, >8000 species) and metal ions endows MPNs with multifunctionality, stimuli‐responsiveness, and customizable physicochemical properties, such as size (ranging from 120 nm to 10 µm) and structure (solid, hollow, porous).^[^ ^21^ ^]^ More important, MPNs avoid the use of exotic nontherapeutic nanocarriers, achieving ultrahigh drug‐loading efficiency (potentially up to 100%), alongside minimal system toxicity and immunogenicity. Additionally, their simple and scalable one‐step nanoprecipitation methods remarkably facilitate subsequent industrial production and clinical translation.^[^ ^22^ ^]^ Nevertheless, the utilization of metal ion coordination with phosphates/phosphonates in nanodrug construction remains infrequent. This is largely attributed to the strong coordination capability of phosphates/phosphonates, which can lead to uncontrollable aggregation with metal ions.^[^ ^23^ ^]^ Although Heck and Ischyropoulou use [ZrO]^2+^ and phosphates/phosphonates as the building blocks to create inorganic‐organic hybrid nanoparticles, [ZrO]^2+^ serves solely as a structural component without any inherent biological activity.^[^ ^6c^ ^]^ Therefore, there is a critical need to develop new approaches for utilizing metal‐coordinated self‐assembly techniques in the development of phosphate/phosphonate‐based nano‐drugs.

The dynamic nature of coordination bonds between metal ions and organic ligands facilitates their modulation through various external stimuli.^[^ ^24^ ^]^ This modulation can be achieved by introducing competitive reagents with superior coordinative capabilities. These reagents can displace the original metal‐organic ligand bonds, initiating a disassembly and reassembly process characterized by bidirectional molecular diffusion flows between the surface and interior of metal‐organic networks. During this stage, the coordination reaction predominantly occurs on the network's surface, leading to the formation of hollow metal‐competitive reagent networks within an association‐dissolution equilibrium. To date, such competitive coordination methods have been adeptly applied to manipulate the structures of MOFs, creating diverse configurations like hierarchical‐pore MOFs, well‐ordered mesoporous MOFs, and geometrical shapes like octahedrons, cuboctahedron, and truncated octahedrons.^[^ ^25^ ^]^ Yet, the exploration of these methods in other coordination‐driven assemblies is still in its infancy. Considering the strong coordination potential of phosphates/phosphonates compared to polyphenols, and the relatively gentle and controllable disassembly and reassembly process, leveraging MPNs as templates with phosphates/phosphonates as competitive agents appears to be a strategic method for producing phosphate/phosphonate‐based nanodrugs with customized morphology and composition.^[^ ^26^ ^]^ This approach taps into the underexplored potential of these coordination‐driven assemblies for drug delivery applications.

**Scheme 1:**
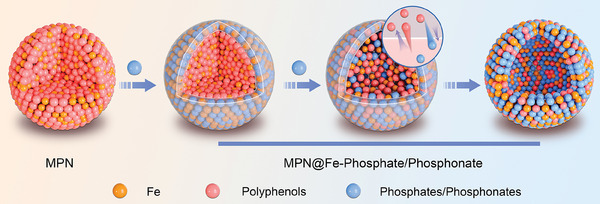
Schematic illustration of nanoengineering of phosphate/phosphonate drugs via competitive coordination with MPNs. Polyphenols: galangin, myricetin, quercetin, baicalein, luteolin, fisetin, apigenin, EGCG, shikonin, etc. Phosphates/phosphonates: zoledronic acid, ATP, ADP, AMP, cGAMP, etc.

Herein, we present a straightforward and universally applicable strategy for preparing phosphate/phosphonate‐based nanodrugs through competitive coordination (**Scheme** **1**). This method employs MPNs as templates and phosphate/phosphonate drugs as competitive agents. By delicately tuning the equilibrium between disrupting existing bonds and establishing new ones via competitive coordination between phenolic ligands, phosphates/phosphonates, and metal ions, we have successfully prepared a variety of hollow nanodrugs with customized compositions and stimuli‐responsiveness. These structures are capable of encapsulating water‐soluble drugs, thereby further enhancing their functionality. For illustration, we utilize Fe^3+^ and galangin (a pyroptosis inducer) to create the MPN template, select zoledronic acid (a U.S. Food and Drug Administration (FDA)‐approved antiresorptive medication) and cyclic guanosine monophosphate‐adenosine monophosphate (cGAMP, a cyclic guanosine monophosphate (GMP)‐adenosine monophosphate (AMP) synthase (cGAS)‐stimulator of interferon genes (STING) signaling pathway (cGAS‐STING) agonist) as competitive agents, and choose doxorubicin hydrochloride (DOX, an FDA‐approved chemotherapeutic agent) as the loaded drug to construct DOX@Fe‐galangin@Fe‐zoledronic acid‐cGAMP (DOX@Fe‐Gal@Fe‐Zol‐cGAMP) nano‐drugs. Both in vitro and in vivo studies reveal that our nanodrugs demonstrate exceptional immunotherapeutic efficacy against breast tumors with lung and bone metastasis, showcasing the potential of our competitive coordination strategy in advancing nanodrug development for complex therapeutic applications.

## 2 Results and Discussion

**Figure 1:**
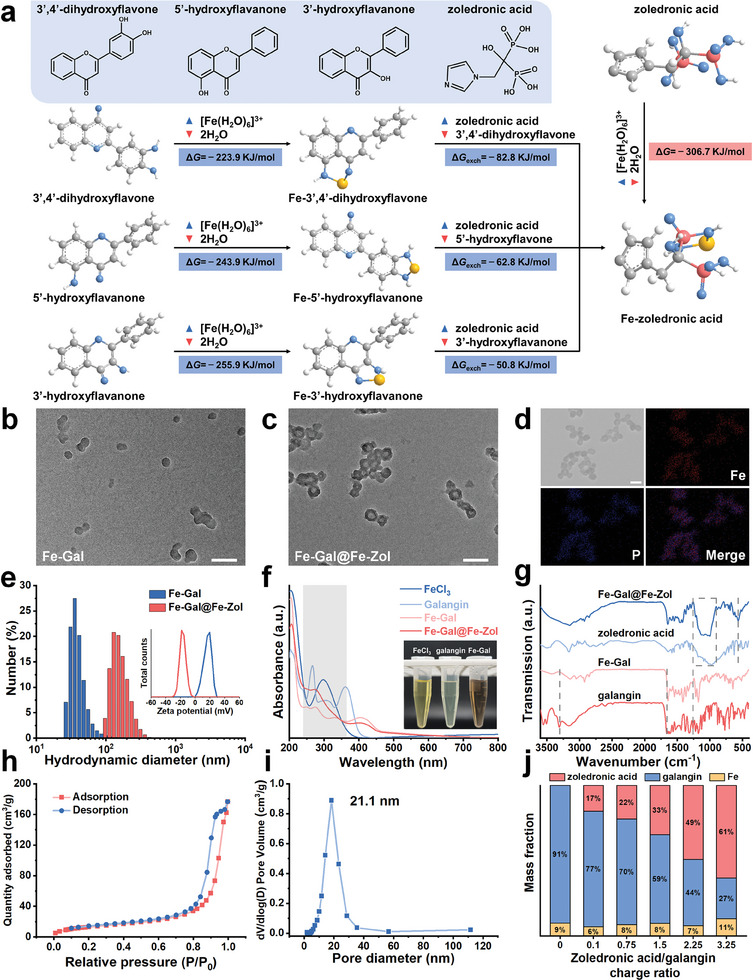
a) Predicted Δ*G* for the coordination of Fe^3+^ to 3′‐hydroxyflavone, 5′‐hydroxyflavone, 3′,4′‐dihydroxyflavone, and zoledronic acid. Δ*G*_exch_ describes the energetics of polyphenols/zoledronic acid ligand exchange. b) TEM image of Fe‐Gal. TEM c) and element mappings d) images of Fe‐Gal@Fe‐Zol. Zoledronic acid/galangin charge ratio is 3.25. Hydrodynamic diameter and zeta potential e), and UV–vis absorption spectra f) of Fe‐Gal and Fe‐Gal@Fe‐Zol. Inset in (f): photographs of FeCl_3_, galangin, and Fe‐Gal solution. g) FTIR spectrum of galangin, Fe‐Gal, zoledronic acid, and Fe‐Gal@Fe‐Zol. N_2_ adsorption/desorption isotherm h) and pore size distribution i) of Fe‐Gal@Fe‐Zol. j) Mass fraction of Fe, galangin, and zoledronic acid in Fe‐Gal@Fe‐Zol obtained at different charge ratios. All scale bars are 100 nm.

Polyphenols typically possess three potential binding sites: acetylacetone, maltol, and catechol, which can selectively coordinate with metal ions.^[^ ^20d^ ^]^ To assess the binding affinity of polyphenols and phosphates/phosphonates toward metal ions, we select 3′‐hydroxyflavone (containing only a maltol group), 5′‐hydroxyflavone (containing only an acetylacetone group), and 3′,4′‐dihydroxyflavone (containing only a catechol group) as representative polyphenols. Zoledronic acid is chosen to represent phosphates/phosphonates (**Figure** **1**a). Using the Gaussian09 program, we systematically calculate the Gibbs free energies (*G*) of Fe^3+^ before and after binding to these ligands. The calculations reveal that the Δ*G* values for Fe^3+^ binding to the maltol, acetylacetone, and catechol groups are −255.9, −243.9, and −223.9 kJ mol^−1^, respectively, which are smaller than the Δ*G* of Fe^3+^ binding to the phosphonate group at −306.7 kJ mol^−1^. Additionally, the predicted energy exchange (Δ*G*_exch_) for Fe^3+^ transfer from the maltol, acetylacetone, and catechol groups to the phosphonate group is −50.8, −62.8, and −82.8 kJ mol^−1^, respectively. These findings demonstrate that the competitive coordination strategy is significantly more thermodynamically favorable, facilitating the exchange of polyphenols and phosphates/phosphonates and the reconfiguration of MPNs.

Then, we experimentally monitor the competitive coordination process using ultraviolet–visible (UV–vis) absorption spectrum and transmission electron microscopy (TEM). The absorption peaks of the benzoyl system of ring‐A in 3′‐hydroxyflavone, 5′‐hydroxyflavone, and 3′,4′‐dihydroxyflavone are red‐shifted from 300–350 to 350–450 nm after the addition of Fe^3+^, indicating their coordination to form MPNs (Figure S1b,d,f, Supporting Information). However, the emergence of the coordination absorption peak of Fe‐zoledronic acid at 250–300 nm and the weakened absorption peak at 300–350 nm after the addition of zoledronic acid imply the replacement of 3′‐hydroxyflavone and 3′,4′‐dihydroxyflavone with zoledronic acid (Figures S1b,d, and S2a, Supporting Information). As Fe‐5′‐hydroxyflavone originally had an absorption peak at 270–275 nm, there is no significant change in the UV–vis absorption spectrum before and after the addition of zoledronic acid (Figure S1f, Supporting Information). However, TEM images show that the spherical‐like MPNs initially formed by Fe^3+^ with 3′‐hydroxyflavone, 5′‐hydroxyflavone, and 3′,4′‐dihydroxyflavone transition to hollow nanostructures after the addition of zoledronic acid, further indicating the competitive coordination by zoledronic acid (Figure S1a,c,e, Supporting Information).

To further explore the universality of this competitive coordination strategy, we select polyphenols with varying coordination modes: single‐modal ((‐)‐epigallocatechin gallate, shikonin, apigenin), two‐modal (baicalein, galangin, luteolin, fisetin), and three‐modal (quercetin, myricetin) coordination sites, as well as phosphates/phosphonates with different numbers of phosphate/phosphonate groups (zoledronic acid, ATP, adenosine diphosphate (ADP), AMP). UV–vis absorption spectra reveal that the cinnamoyl, benzoyl, and butylendiacyl groups of these polyphenols undergo red shifts upon forming MPNs (Figure S3, Supporting Information). The addition of phosphates/phosphonates leads to a significant increase in the absorption peaks at 250–350 nm, indicative of Fe^3+^ coordination with phosphates/phosphonates (Figures S2 and S3, Supporting Information). In the meantime, this addition is also accompanied by a morphological transition from nanospheres to hollow or internally loose nanostructures (Figure S4, Supporting Information). Both the simulation and experimental studies demonstrate that the binding affinity between phosphates/phosphonates and Fe^3+^ is stronger than that between polyphenols and Fe^3+^. This discovery supports a universal competitive coordination strategy, facilitating the preparation of multi‐component nanomedicines using various polyphenols and phosphates/phosphonates.

Subsequently, we utilize Fe^3+^, galangin, and zoledronic acid as building blocks to investigate the dynamic construction process of phosphate/phosphonate‐based nanodrugs. Fe‐galangin (Fe‐Gal) MPNs are first prepared by mixing an aqueous solution of FeCl_3_ and a DMSO solution of galangin at room temperature. The as‐prepared MPNs are monodispersed nanospheres with an average size of 36.1 ± 5.5 nm (Figure 1b). Dynamic light scattering (DLS) measurement indicates an average hydrated diameter of 138.3 ± 15.8 nm with a zeta potential of +16.5 mV (Figure 1e). Unlike the separate solutions of FeCl_3_ and galangin, which are bright yellow, the mixture exhibits a brown color (Figure 1f). This change is attributed to the coordination between the phenolic hydroxyl groups of galangin and Fe^3+^, with a new absorption peak at 602 nm, primarily resulting from charge‐transfer transitions between galangin and Fe^3+^ (Figure 1f). Fourier‐transform infrared (FTIR) spectra further characterize the coordination between galangin and Fe^3+^ (Figure 1g). During MPN formation, the C─OH stretching vibration peak at ≈1258 cm^−1^ decreases, the O─H vibration peak shifts from 3310 to 3425 cm^−1^, and the C═O stretching vibration peak also shifts from 1657 to 1643 cm^−1^, suggesting their coordination with Fe^3+^.

Upon adding zoledronic acid, the resultant Fe‐galangin@Fe‐zoledronic acid (Fe‐Gal@Fe‐Zol, zoledronic/galangin charge ratio is 3.25) hollow nanostructures have an average size of 55.9 ± 10.6 nm and a cavity size of 22.8 ± 6.7 nm (Figure 1c; Figure S5, Supporting Information). The average hydrodynamic diameter increases from 138.3 ± 15.8 to 216.8 ± 48.2 nm, and the zeta potential decreases from +16.5 to −16.9 mV (Figure 1e). The uniform distribution of Fe and P in element mapping images (Figure 1d; Figure S6, Supporting Information), the appearance of P═O and P─O asymmetric stretching vibrations (900–1250 cm^−1^) and P─O bending vibration (560 cm^−1^) in the FTIR spectrum (Figure 1g), as well as the enhancement of absorption at 250–300 nm (Figure 1f), indicate the successful replacement of coordinated galangin by zoledronic acid. N_2_ adsorption/desorption isotherm of Fe‐Gal@Fe‐Zol displays a type IV isotherm with a Brunauer‐Emmett‐Teller (BET) surface area of 49.2 m^2^ g^−1^ (Figure 1h). Accordingly, the cavity volume is 0.26 cm^3^ g^−1^ with the size distribution narrowly centered at 21.1 nm (Figure 1i), consistent with TEM observations.

The influence of zoledronic/galangin charge ratio on the morphology of Fe‐Gal@Fe‐Zol is investigated (Figure S7, Supporting Information). At a zoledronic acid/galangin ratio of 1.5, the internal structure of Fe‐Gal begins to loosen, with this effect becoming more pronounced at the ratio of 2.25. At the ratio of 2.75, cavities start to form, and a small portion of the product exhibits hollow nanostructures. When the ratio reaches 3.25, the majority of the product consists of hollow nanostructures. Although this hollow structure is maintained at the ratio of 3.75, noticeable aggregation of these nanostructures occurs. At the charge ratio of 4.25, severe aggregation takes place, causing the hollow nanostructures to lose stability in aqueous solution. Statistical analysis shows that the cavity size remains unchanged after the charge ratio reaches 3.25 (Figure S7b, Supporting Information). Additionally, the cytotoxicity of Fe‐Gal@Fe‐Zol on tumor cells (4T1 cells) sharply decreases when the zoledronic acid/galangin ratio exceeds 3.25 (Figure S8, Supporting Information). For optimal colloidal stability and cytotoxicity, the zoledronic acid/galangin ratio should therefore remain below 3.25. It is fascinating that the composition of Fe‐Gal@Fe‐Zol can be finely tuned and controlled through this competitive coordination strategy (Figure 1j). With increasing dosage of zoledronic acid from 0 to 3.25 (relative to galangin), its proportion in Fe‐Gal@Fe‐Zol rises from 0% to 61%, while the amount of galangin correspondingly decreases from 91% to 27%.

**Figure 2:**
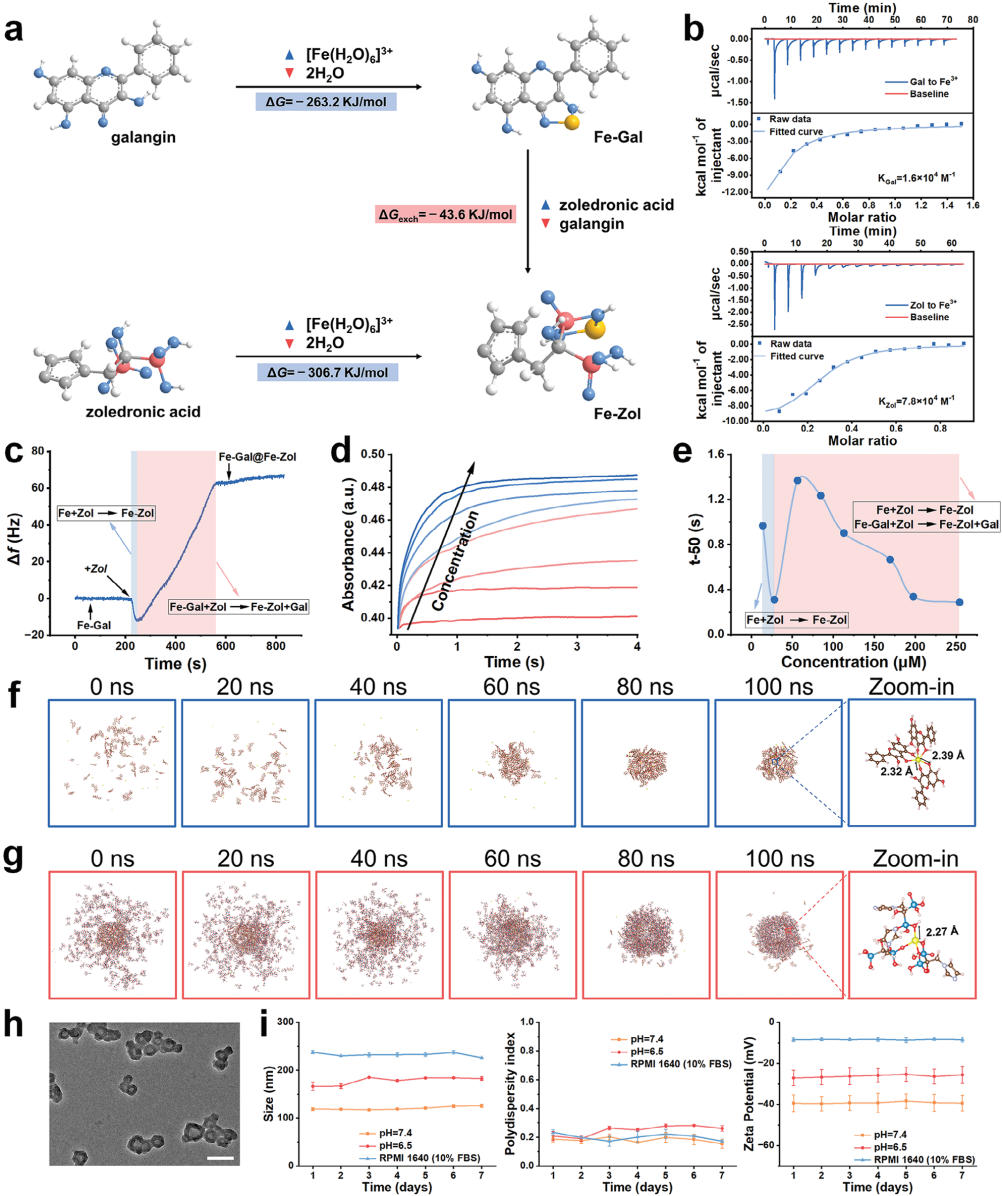
a) Predicted Δ*G* for the coordination of Fe^3+^ to galangin or zoledronic acid. Δ*G*_exch_ describes the energetics of galangin/zoledronic acid ligand exchange. b) ITC analysis of binding affinity between Fe^3+^ and galangin, zoledronic acid. c) Δ*f* traces from QCM monitoring of Fe‐Gal upon the addition of zoledronic acid. d) Concentration‐dependent kinetics of competitive coordination between Fe‐Gal and zoledronic acid. e) t‐50 curve at different zoledronic acid concentrations. f) Snapshots of the self‐assembly process of Fe‐Gal obtained by MD simulations. g) Snapshots of the competitive coordination process between Fe‐Gal and zoledronic acid. h) TEM image of DOX@Fe‐Gal@Fe‐Zol‐cGAMP. Scale bar is 100 nm. i) Temporal evolution of hydrodynamic diameters, PDI, and zeta potential of DOX@Fe‐Gal@Fe‐Zol‐cGAMP storing in water at different pH levels (6.5 and 7.4) and in RPMI 1640 medium with 10% FBS (*n* = 3). Data are shown as mean ± SD; n represents the number of biologically independent samples.

To delve into the competitive coordination process deeply, the binding affinities of Fe^3+^ to galangin and zoledronic acid are quantitatively assessed using isothermal titration calorimetry (ITC) (**Figure** **2**b). The association constant *K* for zoledronic acid (*K*_Zol_ = 7.8 × 10^4^ M^−1^) is determined to be 4.9‐fold higher than that for galangin (*K*_Gal_ = 1.6 × 10^4^ M^−1^). Subsequently, the competitive coordination and binding kinetics between Fe‐Gal MPNs and zoledronic acid are monitored in real‐time by measuring the frequency shift (Δ*f*) via quartz crystal micro‐gravimetry (QCM). As zoledronic acid flows over Fe‐Gal MPNs, a decrease in frequency is observed, indicative of increased mass due to the coordination between coordinatively unsaturated Fe^3+^ and zoledronic acid (Figure 2c). As coordinatively unsaturated Fe^3+^ depleted, zoledronic acid increasingly binds with coordinatively saturated Fe^3+^, intensifying the competitive coordination process. Notably, the incorporation of one bifunctional unit of zoledronic acid displaces two monofunctional units of galangin. Given that the molar mass of zoledronic acid is less than twice that of galangin (*Mr*_Zol_ < 2*Mr*_Gal_), this results in a net decrease in mass, which is reflected as an increase in frequency values. The competitive coordination process under kinetic control is further quantified by monitoring changes in absorption intensity using stopped‐flow experiments (Figure 2d,e). Remarkably, the time required to complete 50% of the coordination process (t‐50) is notably shorter at lower concentrations. This phenomenon is attributed to the preference of zoledronic acid for rapidly coordinating with coordinatively unsaturated Fe^3+^ at these concentrations. As the concentration increases, excess zoledronic acid begins to displace galangin, resulting in a decreased reaction rate, as evidenced by an increase in t‐50. However, further increases in concentration lead to a constant decrease of t‐50. This trend aligns with findings from the QCM test, illustrating the dynamic interplay between reactant concentration and coordination kinetics.

To gain insight into the competitive coordination process, the value of Δ*G* in the Fe‐Gal@Fe‐Zol (zoledronic acid/galangin = 3.25) formation process is calculated (Figure 2a). Consistent with the experimental observations, zoledronic acid exhibits a stronger coordination ability (Δ*G* = −306.7 kJ mol^−1^) compared to galangin (Δ*G* = −263.2 kJ mol^−1^), indicating the thermodynamic feasibility of the competitive coordination process (Δ*G*_exch_ = −43.6 kJ mol^−1^). Molecular dynamic (MD) simulation provides snapshots of the self‐assembly and competitive coordination processes between Fe^3+^, galangin, and zoledronic acid. Fe^3+^ and galangin initially self‐assemble into a spherical MPN (Figure 2f). Upon the introduction of zoledronic acid, it rapidly replaces galangin at the MPN surface due to the competitive coordination effect (Figure 2g). This replacement process continues with the net outflow of galangin, ultimately leading to the formation of a hollow nanostructure. Notably, the magnified images at 100 ns exhibit the clear coordination bonds of galangin (Figure 2f) and zoledronic acid (Figure 2g) with Fe^3+^, further confirming the validity of the competitive coordination effect.

The cavity generated by the competitive coordination process can be used for loading drugs. The drug‐loading capacity of Fe‐Gal@Fe‐Zol hollow nanostructures is evaluated using water‐soluble drug molecules and fluorescent dyes such as DOX, decitabine (DAC), indocyanine green (ICG), and rose bengal (RB) (Figure S9, Supporting Information). TEM images show that the incorporation of these molecules does not compromise the integrity of Fe‐Gal@Fe‐Zol. The emergence of characteristic peaks in both UV–vis absorption and FTIR spectra confirms the successful loading of these molecules. Drug‐loading efficiencies are quantified using the standard curve method, which is calculated to be 12.6% for DOX, 30.2% for RB, 3.9% for IR780, and 28.9% for ICG. These results indicate that Fe‐Gal@Fe‐Zol hollow nanostructures are effective for encapsulating water‐soluble drugs.

To enhance the functionality of Fe‐Gal@Fe‐Zol hollow nanostructures, cGAMP, a cGAS‐STING pathway agonist, is incorporated using the competitive coordination strategy. The competitive coordination and binding kinetics between Fe‐Gal MPNs and cGAMP are assessed using QCM analysis (Figure S10, Supporting Information). As cGAMP flows through Fe‐Gal, a decrease in frequency indicates an increase in mass due to cGAMP loading onto Fe‐Gal MPNs via competitive coordination. Given that a high cGAMP dose (100 µg) can induce IFN‐*β* secretion in normal cells (L929 cells), a final dosage of 50 µg cGAMP is selected for preparing Fe‐Gal@Fe‐Zol‐cGAMP (Figure S11, Supporting Information). Additionally, DOX, a well‐known chemotherapeutic agent, is encapsulated within the cavities. As illustrated in Figure 2h, the eventually adopted DOX@Fe‐Gal@Fe‐Zol‐cGAMP nanodrugs retain the hollow nanostructure with compositions quantified by HPLC and ICP‐AES: 9.7% Fe, 7.8% DOX, 19.5% Gal, 61.8% zoledronic acid, and 1.2% cGAMP (Table S1, Supporting Information). It has been documented that GSH can reduce Fe^3+^ into Fe^2+^, prompting the disassembly of Fe‐Gal@Fe‐Zol‐cGAMP and the release of its components and drugs.^[^ ^27^ ^]^ Consequently, the solution of Fe‐Gal@Fe‐Zol‐cGAMP changes from brown to colorless after co‐incubation with 10 mm GSH, accompanied by the white precipitation of galangin (Figure S12, Supporting Information). This observation is consistent with TEM images that depict the gradual disassembly of Fe‐Gal@Fe‐Zol‐cGAMP into small fragments (Figure S13, Supporting Information). During this disassembly process, the released Fe^2+^ can catalyze the conversion of H_2_O_2_ into hydroxyl radicals (∙OH) via the Fenton reaction. The generation of ∙OH is confirmed by the degradation of methylene blue (MB), marked by a significant decrease in absorbance at 600–700 nm when MB is incubated with H_2_O_2_, GSH, and Fe‐Gal@Fe‐Zol‐cGAMP (Figure S14a, Supporting Information). Simultaneously, the fluorescent band of disodium hydroxyterephthalate (the oxidation product of disodium terephthalate) appears only observed when disodium terephthalate is mixed with Fe‐Gal@Fe‐Zol‐cGAMP, GSH, and H_2_O_2_ (Figure S14b, Supporting Information). The release behavior of DOX from DOX@Fe‐Gal@Fe‐Zol‐cGAMP is further investigated (Figure S15, Supporting Information). In the presence of GSH, the release rate of DOX reaches 85% within 24 h, compared to only 20% in the absence of GSH. These findings demonstrate that the dynamic nature of coordination bonds endow DOX@Fe‐Gal@Fe‐Zol‐cGAMP nanodrugs with ideal GSH‐responsiveness, suggesting potential for targeted release and therapy in tumor microenvironments with high GSH and H_2_O_2_ concentrations.

The colloidal stability of DOX@Fe‐Gal@Fe‐Zol‐cGAMP nanodrugs is assessed by storing them in water at different pH (6.5 and 7.4) and in RPMI‐1640 medium with 10% fetal bovine serum (FBS) for 7 days. Throughout this period, the hydrodynamic diameter, polydispersity index (PDI), and zeta potential of DOX@Fe‐Gal@Fe‐Zol‐cGAMP remain stable without obvious variation (Figure 2i). These results indicate the excellent colloidal stability of DOX@Fe‐Gal@Fe‐Zol‐cGAMP. Notably, in a slightly alkaline environment (pH 7.4), DOX@Fe‐Gal@Fe‐Zol‐cGAMP demonstrates a lower surface potential and smaller hydrodynamic diameters compared to those at pH 6.5. The alkaline environment facilitates the decrease in surface charge, weakening the interactions between the negatively charged nanodrugs and reducing their hydrodynamic diameters. However, in the serum‐containing RPMI‐1640 medium, the hydrodynamic diameters increase due to protein adsorption on the surface. After incubation with FBS, HSA, and BSA, a protein corona inevitably formed around the nanodrugs (Figure S16, Supporting Information).^[^ ^28^ ^]^ This may enhance phagocytic recognition, potentially leading to sequestration and decreased bioavailability of DOX@Fe‐Gal@Fe‐Zol‐cGAMP. PEG‐polyphenol (8‐arm‐PEG‐Dopamine, Mw ≈ 10 kD) is explored as a substitute for small molecule polyphenols to prevent protein adhesion.^[^ ^29^ ^]^ The as‐prepared Fe‐PEG‐Dopamine nanoparticles are monodispersed with an average size of 8.8 ± 1.8 nm (Figure S17a, Supporting Information). Unlike previous findings, no hollow nanostructure is observed after the addition of zoledronic acid (Figure S17b, Supporting Information). The binding affinity between zoledronic acid and Fe‐PEG‐Dopamine is further examined using QCM (Figure S17c, Supporting Information). As zoledronic acid flows through Fe‐PEG‐Dopamine, it rapidly coordinates with unsaturated iron, initiating a competitive coordination process. However, this process progressed slowly, and the final mass remained higher than the initial value. The abundance of phenolic groups makes it difficult for PEG‐polyphenol to detach from Fe‐PEG‐Dopamine, resulting in insufficient competitive coordination. Additionally, studies have shown that anti‐PEG antibodies can accelerate the clearance of systemically administered PEGylated nanodrugs,^[^ ^30^ ^]^ highlighting the need for further research on surface modification of PEG alternatives that mimic PEG's stealth properties.

The cytotoxicity of DOX@Fe‐Gal@Fe‐Zol‐cGAMP toward tumor cells (4T1 cells) and normal cells (L929 cells) is evaluated. Cell viability after different treatments is assessed using the cell counting kit‐8 (CCK‐8) assay (Figure S18, Supporting Information). The results show that all nanoformulations can reduce the viability of 4T1 cells in a concentration‐dependent manner. DOX@Fe‐Gal@Fe‐Zol‐cGAMP exhibits the highest cytotoxicity, killing more than 68% of the cells at a concentration of 100 µg mL^−1^. Calcein AM/PI staining of live/dead cells confirms these findings, with the DOX@Fe‐Gal@Fe‐Zol‐cGAMP group displaying the fewest live cells and most dead cells (Figure S19, Supporting Information). In contrast, the cytotoxicity of nanoformulations toward L929 cells is much lower. More than 83%, 78%, 72%, 77% and 60% of L929 cells survive under the concentration of 100 µg mL^−1^ for Fe‐Gal, Fe‐Gal@Fe‐cGAMP, Fe‐Gal@Fe‐Zol, Fe‐Gal@Fe‐Zol‐cGAMP, and DOX@Fe‐Gal@Fe‐Zol‐cGAMP, respectively. The selective cytotoxicity against tumor cells is attributed to the high intracellular GSH levels, which effectively trigger the disassembly of nanodrugs to release Fe^2+^, galangin, zoledronic acid, cGAMP, and DOX.

**Figure 3:**
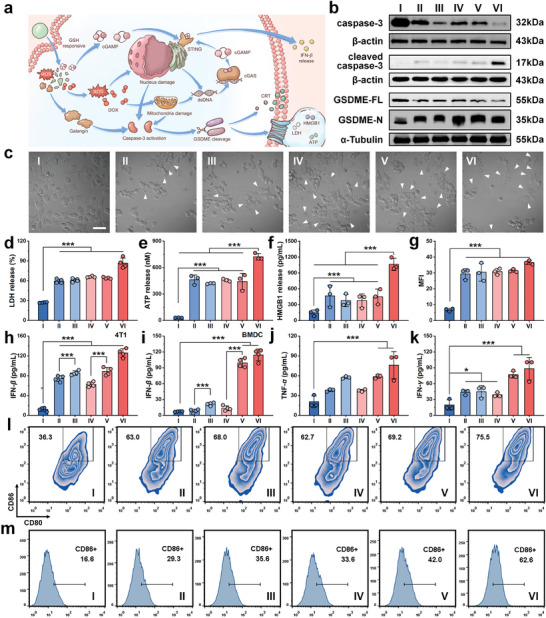
a) Mechanism diagram of immunogenic pyroptosis and STING activation elicited by DOX@Fe‐Gal@Fe‐Zol‐cGAMP nanodrugs. b) Western blot determination of caspase‐3, cleaved caspase‐3, GSDME‐FL, GSDME‐N in 4T1 cells. c) CLSM images (bright field) of 4T1 cells upon different treatments, white allows represented pyroptotic cells with large bubbles. Scale bar is 50 µm. LDH d), ATP e), and HMGB1 f) release levels of 4T1 cells upon different treatments (*n* = 4 in d, *n* = 3 in e and f). g) Mean fluorescence intensity (MFI) values of CRT stain in 4T1 cells upon different treatments (*n* = 3). h) IFN‐*β* secretion levels of 4T1 cells upon different incubation (*n* = 4). IFN‐*β* i), TNF‐*α* j), and IFN‐*γ* k) secretion levels of BMDCs (*n* = 3 in j and k, *n* = 4 in i). l) Representative flow cytometric plots of CD11c^+^CD80^+^CD86^+^ BMDCs in vitro stimulation maturation experiment. m) Representative flow cytometric plots of CD86^+^ M1 macrophages in F40/80^+^ and CD11b^+^ gated BMDMs population in vitro stimulation polarization experiment. Group: (I) Control, (II) Fe‐Gal, (III) Fe‐Gal@Fe‐cGAMP, (IV) Fe‐Gal@Fe‐Zol, (V) Fe‐Gal@Fe‐Zol‐cGAMP, (VI) DOX@Fe‐Gal@Fe‐Zol‐cGAMP. Data are shown as mean ± SD; n represents the number of biologically independent samples. ^*^ *p* < 0.05, ^**^ *p* < 0.01, and ^***^ *p* < 0.001.

To explore the cytotoxic mechanism of DOX@Fe‐Gal@Fe‐Zol‐cGAMP nanodrugs, the morphologies of 4T1 cells after various treatments are observed under confocal microscopy (**Figure** **3**c). Unlike the control group where the cell morphology remains intact, all treatment groups show obvious cell swelling and pronounced membrane bubbles, indicative of the typical morphological characteristics of pyroptosis. A pyroptosis is a proinflammatory form of programmed cell death that depends on the activation of caspase‐3 to cleave gasdermin E (GSDME) into N‐terminal GSDME (GSDME‐N), which forms pores in the cell membrane (Figure 3a).^[^ ^31^ ^]^ Given that reactive oxygen species (ROS) are a prerequisite for pyroptosis,^[^ ^32^ ^]^ and Fe^2+^ can drive ∙OH generation in tumor cells via the Fenton reaction, Fe^2+^ levels within 4T1 cells post‐treatment with various nanoformulations are detected using the FerroOrange probe. As shown in Figure S20 (Supporting Information), all treated groups exhibit bright orange fluorescence, indicating that all nanoformulations disassemble in response to high levels of GSH within 4T1 cells, thereby increasing intracellular Fe^2+^ levels. ROS levels in 4T1 cells are also measured using the DCFH‐DA fluorescence probe (Figure S21, Supporting Information). Mirroring the Fe^2+^ levels, enhanced green fluorescence from intracellular DCF indicates increased ROS levels following treatment with each nanoformulation. Notably, cells treated with DOX@Fe‐Gal@Fe‐Zol‐cGAMP exhibit the highest ROS levels among all groups, likely due to DOX activating nicotinamide adenine dinucleotide phosphate oxidases, which generate superoxide radicals (∙O_2_^−^).^[^ ^33^ ^]^

Since ROS can break DNA, we assess *γ*‐H2AX expression and mitochondrial transmembrane potential (ΔΨm) to quantify DNA damage in cell nuclei and mitochondria. Consequently, all treatments promote *γ*‐H2AX expression and the loss of mitochondrial membrane potential. As a topoisomerase II inhibitor, DOX further suppresses cell proliferation by inducing DNA double‐strand break.^[^ ^34^ ^]^ Therefore, the DOX@Fe‐Gal@Fe‐Zol‐cGAMP group exhibits the strongest fluorescent signals for *γ*‐H2AX (16.7‐fold higher than the control group) and JC‐1 monomer (2.7‐fold higher than the control group), as indicated by the mean fluorescence intensity (MFI) (Figures S22 and S23, Supporting Information). Since DNA damage can activate caspase‐3, which then cleaves GSDME into GSDME‐N to trigger pyroptosis, the key protein expressions of pyroptosis are evaluated via western blot assay. As shown in Figure 3b, Fe‐Gal, Fe‐Gal@Fe‐cGAMP, and Fe‐Gal@Fe‐Zol significantly inhibit caspase‐3 and GSDME expressions but promote cleaved caspase‐3 and GSDME‐N expressions. This effect is attributed not only to ROS but also to the inherent pro‐pyroptosis bioactivity of galangin. The lowest levels of caspase‐3 and GSDME and the highest levels of cleaved caspase‐3 and GSDME‐N are observed in the DOX@Fe‐Gal@Fe‐Zol‐cGAMP group, indicating that DOX further promotes pyroptosis via the caspase‐3/GSDME pathway. Moreover, pretreatment with 2‐bromohexadecanoic acid (BAK/BAX‐Caspase 3‐GSDME pathway inhibitor) and Ac‐DEVD‐CHO (caspase‐3 inhibitor) can enhance the cell viability (Figure S24, Supporting Information). These findings confirm that the caspase‐3/GSDME pathway‐mediated pyroptosis plays a crucial role in the antitumor effects of DOX@Fe‐Gal@Fe‐Zol‐cGAMP.

Pyroptosis is a highly immunogenic mode of cell death, characterized by the substantial expression and release of damage‐associated molecular patterns (DAMPs).^[^ ^35^ ^]^ To validate pyroptosis‐mediated immunogenic cell death (ICD), we measure the release of lactate dehydrogenase (LDH), adenosine triphosphate (ATP), calreticulin (CRT), and high‐mobility group box 1 (HMGB1). The results show a significant increase in LDH and ATP release across all treatment groups, with the addition of DOX leading to the highest level (Figure 3d,e). Furthermore, green immunofluorescence of HMGB1 is observed in the cytoplasm after being treated with various nanoformulations (Figure S25, Supporting Information). Most of HMGB1 is released from the nucleus after treatment with DOX@Fe‐Gal@Fe‐Zol‐cGAMP, with the HMGB1 signal in the nucleus of some cells disappearing entirely. The levels of HMGB1 in 4T1 cell culture medium after various treatments are further quantified using the ELISA kit. The concentration of HMGB1 in the medium is mildly increased after treatment with Fe‐Gal, Fe‐Gal@Fe‐cGAMP, Fe‐Gal@Fe‐Zol, and Fe‐Gal@Fe‐Zol‐cGAMP, while it is highest after DOX@Fe‐Gal@Fe‐Zol‐cGAMP treatment, showing a 7.3‐fold increase compared to the control group (Figure 3f). Similarly, the cell‐surface exposure of CRT is enhanced after various treatments, with the DOX@Fe‐Gal@Fe‐Zol‐cGAMP group exhibiting the brightest green immunofluorescence of CRT on the cell membrane, which is 5.4‐fold higher compared to the control group (Figure 3g; Figure S26, Supporting Information). In summary, DOX@Fe‐Gal@Fe‐Zol‐cGAMP induced 4T1 cell death has the main characteristics of pyroptosis‐mediated ICD.

cGAS‐STING pathway is critical for initiating antitumor immunity.^[^ ^36^ ^]^ Activation of the cGAS‐STING pathway involves the recognition of double‐stranded DNA (dsDNA) molecules by cGAS, which then catalyzes the synthesis of cyclic dinucleotides such as cGAMP to activate STING and produce type‐I interferon (IFN‐*β*) (Figure 3a). These molecules further stimulate the dendritic cell (DC) maturation, antigen cross‐presentation, effector T cell activation and infiltration, and the reversal of the tumor immunosuppressive microenvironments, all of which are essential for effective antitumor immunotherapy.^[^ ^37^ ^]^ Since ROS and DOX can cause DNA damage, facilitating the rapid cytosolic release of DNA fragments, dsDNA in the cytoplasm is monitored using Picogreen. An enhanced green signal is detected post‐treatment with all nanoformulations, indicating the release of dsDNA into the cytoplasm (Figure S27, Supporting Information). IFN‐*β* secretion in 4T1 cells treated with various nanoformulations is measured. Due to the ROS‐induced release of dsDNA, the secretion levels of IFN‐*β* after Fe‐Gal and Fe‐Gal@Fe‐Zol treatments are 6.2‐fold and 5.2‐fold higher than that in the control group (Figure 3h). Loading with cGAMP further increased IFN‐*β* secretion, showing 7.2‐fold (Fe‐Gal@Fe‐cGAMP) and 7.4‐fold (Fe‐Gal@Fe‐Zol‐cGAMP) increases compared to the control group. The highest level of IFN‐*β* is observed in the DOX@Fe‐Gal@Fe‐Zol‐cGAMP group, with a 10.6‐fold increase over the control group. After pretreatment with inhibitors that competitively bind to STING (SN‐011 and STING‐IN‐2), IFN‐*β* secretion induced by DOX@Fe‐Gal@Fe‐Zol‐cGAMP is significantly reduced (Figure S28, Supporting Information). This reduction in IFN‐*β* secretion due to the blockage of cGAMP binding to STING suggests that DOX@Fe‐Gal@Fe‐Zol‐cGAMP‐induced IFN‐*β* secretion is dependent on cGAMP's activation of the cGAS‐STING pathway. These results indicate that the combination of ROS, cGAMP, and DOX efficiently activates the cGAS‐STING pathway and promotes the release of type I interferon in tumor cells.

To analyze the effect of released DAMPs and IFN‐*β* on DC maturation, 4T1 cells are first co‐incubated with various nanoformulations for 24 h. The supernatants are then collected and added to the suspension of murine bone marrow‐derived dendritic cells (BMDCs). The expression of the mature marker (CD11c^+^CD80^+^CD86^+^) on BMDCs is determined by flow cytometry. Compared to the control group, all nanoformulations can increase the proportion of mature BMDCs, with the DOX@Fe‐Gal@Fe‐Zol‐cGAMP group showing the highest level of maturation (Figure 3l). This indicates that DAMPs and IFN‐*β* released from 4T1 cells can efficiently promote BMDCs maturation. Moreover, the cGAS‐STING pathway in BMDCs can be further activated after engulfing these released DAMPs and IFN‐*β*. Unlike Fe‐Gal and Fe‐Gal@Fe‐Zol, which only slightly increase the secretion of IFN‐*β* in BMDCs, Fe‐Gal@Fe‐cGAMP and Fe‐Gal@Fe‐Zol‐cGAMP significantly boost IFN‐*β* secretion in BMDCs (Figure 3i). This suggests that the loaded cGAMP in nanodrugs is released from dying 4T1 cells and internalized by BMDCs for the cGAS‐STING pathway activation. DOX further promotes the release of cGAMP by enhancing pyroptosis, resulting in the highest IFN‐*β* secretion in the DOX@Fe‐Gal@Fe‐Zol‐cGAMP group. Along with the maturation and cGAS‐STING pathway activation, the secretion of pro‐inflammatory cytokines (TNF‐*α*, IFN‐*γ*) in BMDCs exhibits a similar trend to IFN‐*β* (Figure 3j,k).

Zoledronic acid can further promote tumor‐associated macrophage (TAM) polarization from the pro‐tumorigenic M2 phenotype to the tumoricidal M1 phenotype.^[^ ^38^ ^]^ The phenotypes of macrophages after different treatments are evaluated using the same procedure as for BMDC maturation, except for replacing BMDCs with murine bone marrow‐derived macrophages (BMDMs). Compared to the control group, Fe‐Gal increases the proportion of M1 BMDMs (CD11b^+^F4/80^+^CD86^+^) from 16.6% to 29.3% due to tumor cell pyroptosis (Figure 3m). This value further increases to 35.6%, 33.6%, and 42.0% in the Fe‐Gal@Fe‐cGAMP, Fe‐Gal@Fe‐Zol, and Fe‐Gal@Fe‐Zol‐cGAMP groups, respectively, attributed to the activation of the cGAS‐STING pathway and the inherent pro‐polarization bioactivity of zoledronic acid. Benefiting from the stimulation of DOX on pyroptosis and the activation of the cGAS‐STING pathway, DOX@Fe‐Gal@Fe‐Zol‐cGAMP cause the highest proportion of M1 BMDMs (62.6%) across all the groups.

**Figure 4:**
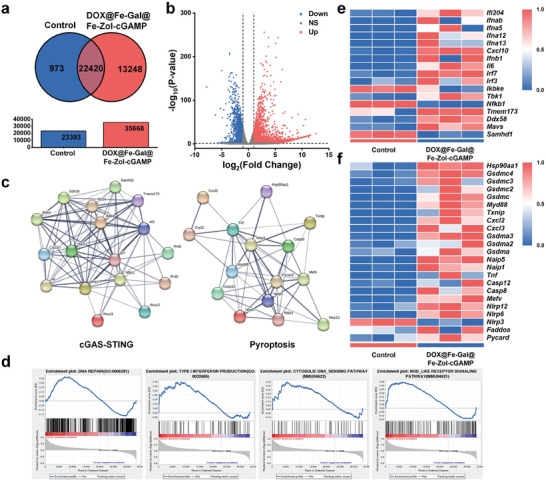
RNA sequencing analysis. a) Venn diagram of all expressed genes in each group. b) Volcano plot of the distributions of differentially expressed genes after DOX@Fe‐Gal@Fe‐Zol‐cGAMP treatment (|log_2_ (Fold Change)| > 1 and *p*‐value < 0.05). c) Protein‐protein interaction network from the STRING database. d) GSEA analysis of differentially expressed genes that hit the DNA repair, type I interferon production, cytosolic DNA‐sensing pathway, and NOD‐like receptor signaling pathway gene sets. Heat‐map analysis of mRNA expression levels of genes involved in cGAS‐STING pathway e) and pyroptosis f).

To delve deeper into the cytotoxic mechanism of DOX@Fe‐Gal@Fe‐Zol‐cGAMP nanodrugs, we conduct gene expression analysis in post‐treatment 4T1 cells using RNA sequencing. The Venn diagram illustrates that 13248 genes are transcribed after DOX@Fe‐Gal@Fe‐Zol‐cGAMP treatment (**Figure** **4**a). The volcano plot reveals 7625 differentially expressed genes (|log_2_ (Fold Change)| > 1 and *p*‐value <0.05) between treated and untreated cells, including 5743 upregulated and 1882 downregulated genes (Figure 4b), indicating that DOX@Fe‐Gal@Fe‐Zol‐cGAMP has a noticeable impact on gene expression at the cellular level. Gene set enrichment analysis (GSEA) indicates significant upregulation of pathways such as the cGAS‐STING pathway (cytosolic DNA‐sensing pathway), pyroptosis (via the NOD‐like receptor signaling pathway), DNA repair, and type I interferon production (Figure 4d). Further expression analysis focused on genes related to pyroptosis and the cGAS‐STING pathway reveals upregulation of relevant mRNAs and a marked decrease in the expression of inhibitory genes (Figure 4e,f). The network of protein‐protein interactions among differentially expressed genes indicates close associations, underscoring their collective impact on cellular processes (Figure 4c). These findings confirm that DOX@Fe‐Gal@Fe‐Zol‐cGAMP nano drugs not only effectively trigger pyroptosis but also activate the cGAS‐STING pathway.

Encouraged by its outstanding antitumor performance in vitro, we explore the in vivo antitumor efficacy of DOX@Fe‐Gal@Fe‐Zol‐cGAMP nanodrugs in 4T1 tumor‐bearing mice. Due to the fluorescence interference of DOX for in vivo imaging, we use indocyanine green (ICG)‐labeled Fe‐Gal@Fe‐Zol‐cGAMP (*^ICG^* Fe‐Gal@Fe‐Zol‐cGAMP) to characterize the bio‐distribution and circulating half‐life of the nanodrugs. After intravenous injection, the fluorescence signal of *^ICG^* Fe‐Gal@Fe‐Zol‐cGAMP is initially detected at the tumor site after 2.5 h, increasing gradually and peaking 24 h postinjection, with noticeable signals persisting even after 48 h (Figure S29a, Supporting Information). In contrast, the fluorescence signal from free ICG is also detectable in tumors but is comparatively weaker and diminished more rapidly, highlighting non‐specific distribution and faster systemic clearance. Ex vivo fluorescence imaging of major organs and tumors confirms that *^ICG^* Fe‐Gal@Fe‐Zol‐cGAMP exhibits the strongest fluorescence signal within the tumor at 48 h, whereas the fluorescence from free ICG predominantly accumulated in the liver and kidneys (Figure S29b, Supporting Information). Additionally, the circulating half‐life of *^ICG^* Fe‐Gal@Fe‐Zol‐cGAMP is calculated to be 136.3 ± 2.5 min, significantly exceeding that of free ICG (40.7 ± 7.5 min) (Figure S30, Supporting Information). These results indicate that Fe‐Gal@Fe‐Zol‐cGAMP is effectively concentrated within tumor tissues, enhancing its therapeutic efficacy and potential for targeted tumor treatment.

**Figure 5:**
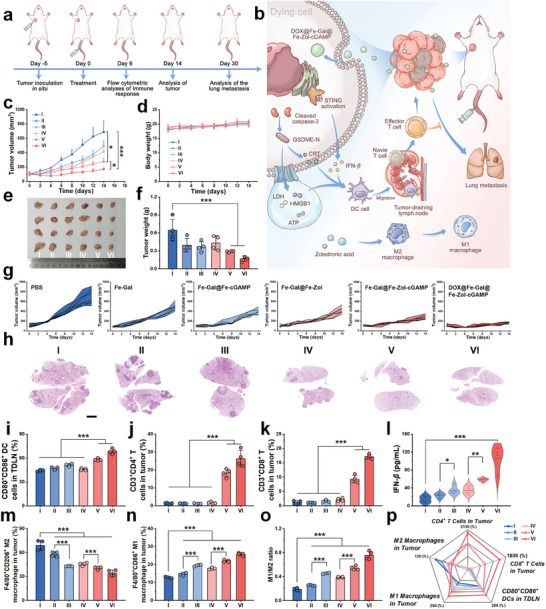
In vivo antitumor study. a) Schematic illustration of the treatment protocol in orthotopic 4T1 tumor model. b) Schematic illustration showing the mechanism for the immune activation and tumor metastasis inhibition at the tumor site. Volumes of tumors c) and weights d) of mice in each group throughout the treatment period (*n* = 7). Photographs e) and weights f) of tumors after different treatments (*n* = 4). g) Individual tumor growth kinetics in different groups (*n* = 7). h) H&E staining of lung tissues at 30 days after treatment. Scale bar is 2 mm. i) Quantitative analysis of matured DCs (CD11c^+^CD80^+^CD86^+^) in TDLNs after different treatments by flow cytometry (*n* = 3). Quantitative analysis of Ths j) and CTLs k) in tumors after different treatments by flow cytometry (*n* = 3). l) IFN‐*β* secretion levels in the serum of 4T1 tumor‐bearing mice after different treatments (*n* = 4). m) Quantitative analysis of M1‐phenotype macrophages (F4/80^+^CD86^+^) and n) M2‐phenotype macrophages (F4/80^+^CD206^+^) in tumors after different treatments by flow cytometry (*n* = 4). o) The ratio of M1/M2 in the tumors after different treatments (*n* = 4). p) Summary of CTLs, Ths, M1 macrophages, and M2 macrophages in tumor tissues and DCs in TDLNs (*n* = 4). Group: (I) Control, (II) Fe‐Gal, (III) Fe‐Gal@Fe‐cGAMP, (IV) Fe‐Gal@Fe‐Zol, (V) Fe‐Gal@Fe‐Zol‐cGAMP, (VI) DOX@Fe‐Gal@Fe‐Zol‐cGAMP. Data are shown as mean ± SD; n represents the number of biologically independent samples. ^*^ *p* < 0.05, ^**^ *p* < 0.01, and ^***^ *p* < 0.001.

Subsequently, we establish an orthotopic 4T1 breast tumor model to evaluate the antitumor effects of DOX@Fe‐Gal@Fe‐Zol‐cGAMP nanodrugs (**Figure** **5**a). When tumor volumes reach ≈100 mm^3^, mice are randomly divided into 6 groups: I: Control; II: Fe‐Gal; III: Fe‐Gal@Fe‐cGAMP; IV: Fe‐Gal@Fe‐Zol; V: Fe‐Gal@Fe‐Zol‐cGAMP; VI: DOX@Fe‐Gal@Fe‐Zol‐cGAMP. Each group receives intravenous administrations of various formulations at doses equivalent to those in group VI. Tumor volume and body weight changes are recorded every other day. The tumor growth curves show that all treated groups exhibit varying degrees of tumor growth inhibition compared to the rapid tumor growth in the control group. By day 14, the inhibition rate in groups II–VI is 28.7%, 39.9%, 38.8%, 59.8%, and 75.1%, respectively (Figure 5c,g). Photographs and weights of ex vivo tumors confirm the same trend, highlighting the efficacy of these nanoformulations in suppressing tumor growth (Figure 5e,f). H&E staining shows that there is obvious necrosis in tumors after Fe‐Gal@Fe‐Zol‐cGAMP and DOX@Fe‐Gal@Fe‐Zol‐cGAMP treatments (Figure S31, Supporting Information). Immunofluorescence (IF) analysis is further performed to explore the antitumor mechanism of DOX@Fe‐Gal@Fe‐Zol‐cGAMP (Figure S32, Supporting Information). The concentration of IFN‐*β* increases in the serum of mice after DOX@Fe‐Gal@Fe‐Zol‐cGAMP treatment, which is 7.4‐fold higher than that of the control group (Figure 5l). The fluorescence signals of cleaved caspase‐3, GSDME‐N, and IFN‐*β* can be observed in all treated groups, with the highest fluorescence intensity in group VI, suggesting that DOX@Fe‐Gal@Fe‐Zol‐cGAMP can efficiently cause pyroptosis and activate the cGAS‐STING pathway in tumors.

To assess the benefits of nanoformulations, we compare the antitumor effects of DOX@Fe‐Gal@Fe‐Zol‐cGAMP nanodrugs and the mixture of each ingredient at an equivalent dosage. Although the mixture also inhibits tumor growth, it leads to a sharp decline in the body weight of the mice, with none surviving beyond 8 days (Figure S33, Supporting Information). This adverse outcome is attributed to the poor water solubility of galangin and the potent side effects associated with zoledronic acid and DOX. In contrast, mice treated with nanodrugs experienced only a slight decrease in body weight following administration, which subsequently recovered. These findings demonstrate that DOX@Fe‐Gal@Fe‐Zol‐cGAMP nanodrugs not only retain the bioactivities of the ingredients but also mitigate their toxicities.

The combination of pyroptosis inducers and cGAS‐STING agonists in the design of nanomedicines aims to stimulate DC maturation, promote cytotoxic T lymphocyte (CTL) infiltration, and accelerate TAM repolarization, thereby enhancing tumor immunogenicity and reversing immunosuppressive tumor microenvironments (Figure 5b). Flow cytometry is used to evaluate these immunological responses in tumor tissues. Compared to other groups, the proportion of mature DCs in tumor‐draining lymph nodes (TDLNs) increases from 44.3% to 58.1% and 68.3% in groups V and VI, respectively (Figure 5i; Figure S34, Supporting Information). This is likely due to the synergistic effect of pyroptosis and cGAS‐STING pathway activation. Since DC maturation is crucial for effector T cell differentiation, the CTL (CD3^+^CD8^+^) proportion in tumors increase from 1.1% to 9.3% and 17.2%, and the T helper cells (Ths, CD3^+^CD4^+^) proportion in tumors increase from 1.4% to 18.3% and 26.4% after Fe‐Gal@Fe‐Zol‐cGAMP and DOX@Fe‐Gal@Fe‐Zol‐cGAMP treatments (Figure 5j,k; Figure S35, Supporting Information). These findings are corroborated by immunofluorescence staining, which shows obvious fluorescence signals of CTLs and Ths in groups V and VI (Figure S36, Supporting Information). Additionally, all the nanoformulations foster TAM repolarization to some extent, attributed to the immunomodulatory effects of pyroptosis and the cGAS‐STING pathway. As expected, DOX@Fe‐Gal@Fe‐Zol‐cGAMP caused a significant increase in the relative number of M1 macrophages (F4/80^+^CD206^+^) and the substantial reduction of M2 macrophages (F4/80^+^CD86^+^), leading to the highest M1/M2 ratio across all the groups (Figure 5m–o; Figure S37, Supporting Information). Overall, DOX@Fe‐Gal@Fe‐Zol‐cGAMP nanodrugs can effectively improve DC maturation, CTL infiltration, and TAM repolarization, thereby enhancing antitumor immunity and ameliorating the immunosuppressive microenvironments in tumors (Figure 5p).

To assess the capability of DOX@Fe‐Gal@Fe‐Zol‐cGAMP nanodrugs against tumor metastasis, the lungs of mice in each group are collected and analyzed 30 days post‐treatment. Photographs in Figure S38 (Supporting Information) reveal that lung metastases are reduced in all treatment groups, with the DOX@Fe‐Gal@Fe‐Zol‐cGAMP treated mice exhibiting the fewest metastatic nodules. H&E staining shows extensive tumor metastases in the control group, whereas only a few small metastases are present in the lungs of mice treated with DOX@Fe‐Gal@Fe‐Zol‐cGAMP (Figure 5h).

**Figure 6:**
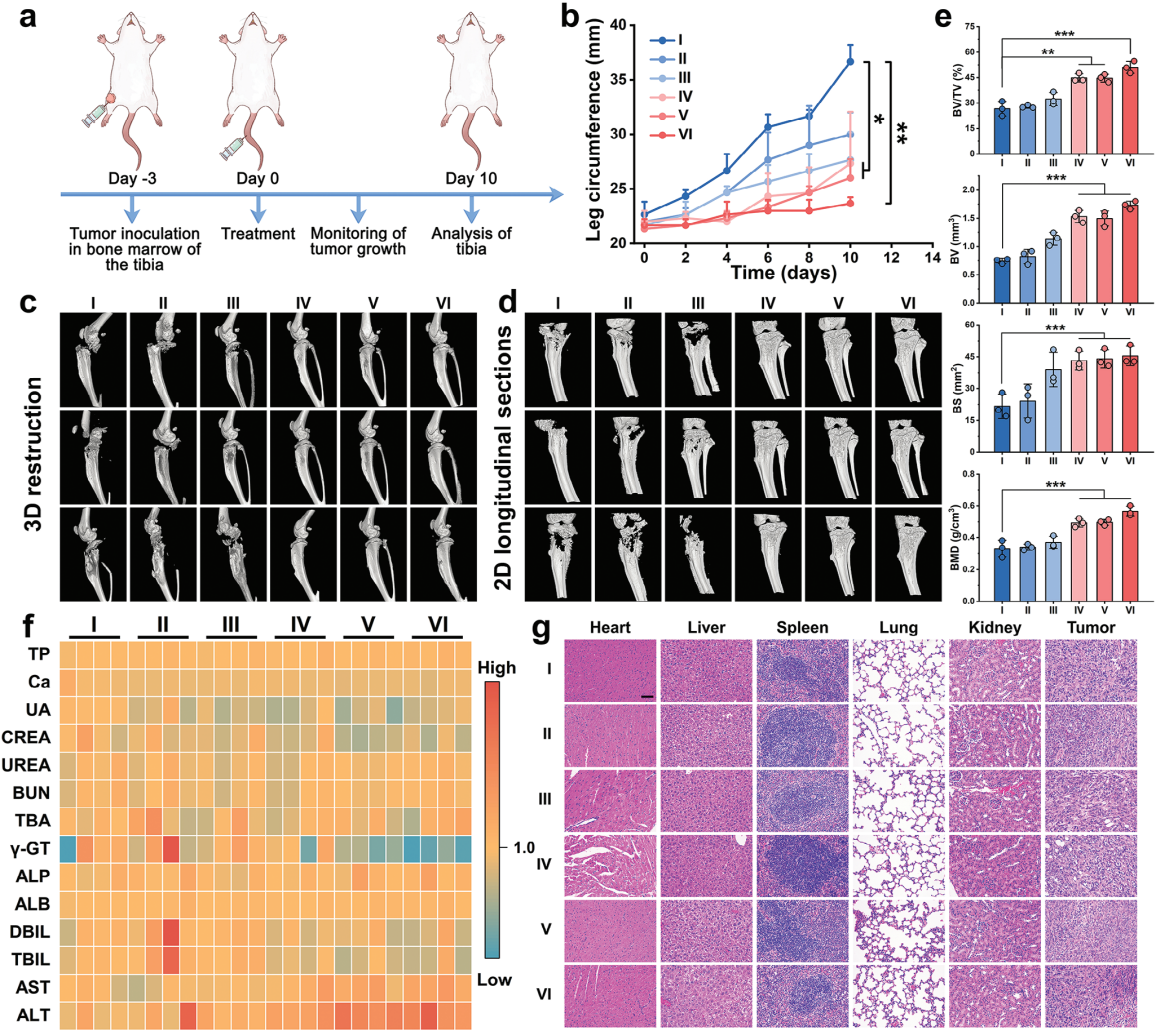
Therapeutic effects of DOX@Fe‐Gal@Fe‐Zol‐cGAMP nanodrugs against early bone metastasis. a) Schematic illustration of the treatment protocol in 4T1 tumor model of breast cancer bone metastasis. b) Circumference changes of tumor‐bearing legs during the treatments (*n* = 3). 3D micro‐CT reconstruction c) and 2D longitudinal sections images (c) of tibias after different treatments. e) BV, BV/TV, BS, and BMD analyses based on micro‐CT after different treatments (*n* = 3). f) Main indexes of liver and renal functions analysis of mice upon various treatments. g) H&E‐stained images of organs collected from mice at the end of different treatments. Scale bar is 100 µm. Group: (I) Control, (II) Fe‐Gal, (III) Fe‐Gal@Fe‐cGAMP, (IV) Fe‐Gal@Fe‐Zol, (V) Fe‐Gal@Fe‐Zol‐cGAMP, (VI) DOX@Fe‐Gal@Fe‐Zol‐cGAMP. Data are shown as mean ± SD; n represents the number of biologically independent samples. ^*^ *p* < 0.05, ^**^ *p* < 0.01, and ^***^ *p* < 0.001.

Since zoledronic acid is a well‐known clinical drug for treating osteolytic bone metastases in malignant tumors, we establish a 4T1 cell‐derived bone metastasis mouse model via intratibial injection of 4T1 cells (**Figure** **6**a). 3 days post‐injection, mice are randomized into six groups and received intravenous injections of I: Control, II: Fe‐Gal, III: Fe‐Gal@Fe‐cGAMP, IV: Fe‐Gal@Fe‐Zol, V: Fe‐Gal@Fe‐Zol‐cGAMP, and VI: DOX@Fe‐Gal@Fe‐Zol‐cGAMP. The circumference of the inoculated legs is measured every other day (Figure 6b). Mice in group I display the greatest increase in leg circumference, indicative of uncontrolled tumor growth. Fe‐Gal and Fe‐Gal@Fe‐cGAMP can mildly limit bone metastases, resulting in smaller leg circumferences compared to the control group. Benefiting from the inherent anti‐bone resorption bioactivity of zoledronic acid, Fe‐Gal@Fe‐Zol, and Fe‐Gal@Fe‐Zol‐cGAMP show stronger inhibition against tumor growth. Mice treated with DOX@Fe‐Gal@Fe‐Zol‐cGAMP show the smallest leg circumference, attributable to the additional effect of DOX in curbing tumor cell proliferation.

Micro‐computed tomography (micro‐CT) scanning is performed to evaluate the tibia destruction caused by bone metastases. 3D micro‐CT reconstructions and 2D longitudinal section photographs reveal that the inclusion of zoledronic acid (groups IV–VI) significantly reduces tibial damage compared to the other groups, with the tibias in DOX@Fe‐Gal@Fe‐Zol‐ cGAMP group exhibiting the maximum intactness (Figure 6c,d). Accordingly, key metrics such as bone volume (BV), bone volume/total volume (BV/TV), bone surface (BS), and bone mineral density (BMD) notably increase in groups IV–VI, with group VI exhibiting the most pronounced inhibitory effect against osteolysis driven by tumors (Figure 6e). Together, DOX@Fe‐Gal@Fe‐Zol‐cGAMP nanodrugs not only exhibit potent cytotoxicity and accurate selectivity in eradicating orthotopic breast tumors but also activate an antitumor immune response against lung and bone metastasis.

Finally, the biosafety of DOX@Fe‐Gal@Fe‐Zol‐cGAMP nanodrugs is evaluated. Throughout the treatment period, no significant weight loss is observed in any group (Figure 5d). At the end of the treatment, serum samples from mice in each group are tested for liver and kidney function. All indices are within the normal range, except for the uric acid levels, which are elevated (Figure 6f). This elevation is thought to be caused by the accelerated metabolism of tumor cells. The main organs (heart, liver, spleen, lungs, and kidneys) are analyzed using H&E staining. As illustrated in Figure 6g, no evident organ damage is observed in mice across all groups. These results suggest that the treatment does not induce significant systemic toxicity, confirming the high biosafety of DOX@Fe‐Gal@Fe‐Zol‐cGAMP nanodrugs.

## 3 Conclusion

In summary, we have demonstrated the nanoengineering of phosphate/phosphonate drugs through a competitive coordination strategy. By leveraging the difference in coordination capabilities between polyphenols and phosphates/phosphonates with metal ions, we can integrate phosphates/phosphonates with varying biological activities and molecular structures into nanodrugs with customized composition. This nanoengineering method not only improves the biocompatibility and bioavailability of phosphates/phosphonates without compromising their biological activities but also enables their stimuli‐responsive release in pathological microenvironments. Following this competitive coordination strategy, we develop DOX@Fe‐Gal@Fe‐Zol‐cGAMP nanodrugs. The dynamic nature of the coordination bonds endows these nanodrugs with ideal GSH responsiveness for targeted release and therapy in tumor microenvironments. Both in vitro and in vivo studies reveal that by triggering pyroptosis and activating the cGAS‐STING pathway, our nanodrugs exhibit potent cytotoxicity and accurate selectivity in eradicating orthotopic breast tumors. Additionally, they activate an antitumor immune response against lung and bone metastasis. Because our competitive coordination strategy is universal for a variety of phosphate/phosphonate agents, it holds significant potential for promoting the clinical efficacy of phosphate/phosphonate drugs and advancing nanodrug development for complex therapeutic applications.

## 4 Experimental Section

### Animals

Female BALB/c mice (6–8 weeks) were purchased from Charles River Laboratories (Beijing, China). All mice were housed in the specific pathogen‐free, temperature‐controlled facility at the Institute of Translational Medicine, The First Hospital of Jilin University, Changchun, China. The animal experiments were carried out according to the protocol approved by the Ministry of Health in the People's Republic of P. R. China. Minimization of experimental animal numbers and experimental protocols were approved by the Ethics Committee of the First Hospital of Jilin University, Changchun, P. R. China (approval number: 20200693).

### Preparation of Formulations

The 0.8 mL of FeCl_3_·6H_2_O solution (20 mg mL^−1^, H_2_O) was added to 40 mL of deionized water, and 0.8 mL of galangin solution (5 mg mL^−1^, DMSO) was added with magnetic stirring at 600 rpm. The mixture was centrifuged at 18000 rpm for 50 min to collect the supernatant and the precipitate was re‐dispersed in 1 mL of deionized water. This centrifugation step needs to be repeated once. The final solution is the Fe‐Gal nanodrug aqueous solution (1 mg mL^−1^).

The 0.6 mL of FeCl_3_·6H_2_O solution (20 mg mL^−1^, H_2_O) was added to 30 mL of deionized water, and 0.6 mL of galangin (5 mg mL^−1^, DMSO) was added with magnetic stirring at 600 rpm. Subsequently, 5.1 mL of zoledronic acid aqueous solution (2 mg mL^−1^) was added. The mixture was centrifuged at 8800 rpm for 10 min to collect the supernatant and the precipitate was re‐dispersed in 1 mL of deionized water. This centrifugation step needs to be repeated two times. The final solution is Fe‐Gal@Fe‐Zol nanodrug aqueous solution (1 mg mL^−1^, zoledronic acid/galangin charge molar ratio = 3.25). On this basis, Fe‐Gal@Fe‐Zol with charge ratios of 0.1, 0.75, 1.5, and 2.25 were obtained by only changing the amount of zoledronic acid solution to 0.2, 1.2, 2.4, and 3.6 mL.

### Statistical Analysis

The data from the heatmap (Figures 4e,f and 6f) is pre‐normalized. All data with error bars are presented as the mean ± standard deviation (SD). n represents the number of biologically independent samples. Statistical significance was calculated by using one‐way ANOVA, assigned at ^*^ *p* < 0.05, ^**^ *p* < 0.01, ^***^ *p* < 0.001. All the graphing and statistical analysis were carried out using Origin 2024b. Mean fluorescence intensity statistics were performed using Image J.

## Conflict of Interest

The authors declare no conflict of interest.

## Supporting information



Supporting Information

## Data Availability

The data that support the findings of this study are available from the corresponding author upon reasonable request.
