# Supplementary material for: Nanoengineering of Phosphate/Phosphonate Drugs via Competitive Replacement with Metal‐Phenolic Networks to Overcome Breast Tumor with Lung and Bone Metastasis
Source: Adv Sci (Weinh). 2024 Nov 18;12(2):2413201. doi: 10.1002/advs.202413201 (PMC11727113; doi:10.1002/advs.202413201)
Supplement: Supplementary file 1 — Supporting Information [file ADVS-12-2413201-s001.pdf]

ADVANCED SCIENCE
Open Access

Supporting Information

for *Adv. Sci.*, DOI 10.1002/advs.202413201

Nanoengineering of Phosphate/Phosphonate Drugs via Competitive Replacement with Metal-Phenolic Networks to Overcome Breast Tumor with Lung and Bone Metastasis

Wanrui Shi, Dashuai Liu, Wenjie Feng, Yang Chen, Yonggang Wang, Zhihong Nie*, Yi Liu* and Hao Zhang**

Supporting information

Nanoengineering of Phosphate/Phosphonate Drugs via Competitive Replacement with Metal-Phenolic Networks to Overcome Breast Tumor with Lung and Bone Metastasis

Wanrui Shi,[1,2] Dashuai Liu,[2] Wenjie Feng,[1] Yang Chen,[2] Yonggang Wang,[3,*] Zhihong Nie,[4,*] Yi Liu,[1,2,*] and Hao Zhang[1,2,*]

[1]Joint Laboratory of Opto-Functional Theranostics in Medicine and Chemistry, Institute of Translational Medicine, The First Hospital of Jilin University, Changchun 130021, P. R. China.

[2]State Key Laboratory of Supramolecular Structure and Materials, College of Chemistry, Jilin University, Changchun 130012, P. R. China

[3]Department of Cardiovascular Centre, The First Hospital of Jilin University, Changchun 130021, P. R. China

[4]State Key Laboratory of Molecular Engineering of Polymers, Department of Macromolecular Science, Fudan University, Shanghai 200438, P. R. China

*Address correspondence to wangyg1982@jlu.edu.cn; znie@fudan.edu.cn; yiliuchem@jlu.edu.cn; hao_zhang@jlu.edu.cn

Experimental Section

Materials Iron chloride hexahydrate ($FeCl_3 \cdot 6H_2O$), Zoledronic Acid, Doxorubicin hydrochloride, Dimethyl sulfoxide (DMSO), disodium terephthalate (DT), methylene blue (MB), Shikonin, Baicalein, Apigenin, Myricetin, Quercetin, Luteolin, Fisetin, (-)-Epigallocatechin gallate (EGCG), IR780, Decitabine, and Indocyanine Green (ICG) were purchased from Aladdin Industrial Corporation (Shanghai, China). Hydrogen peroxide (H_2O_2) was purchased from Beijing Chemical Works. 2’,3’-cGAMP was purchased from Med-bio Medical Technology Co., Ltd. Cell counting kit-8 (CCK-8) was purchased from Bimake. FerroOrange (F374) was purchased from Dojindo. Hoechst 33342 staining solution for live cells, LDH Cytotoxicity Assay Kit, Reactive oxygen species (ROS) assay kit, Recombinant murine GM-CSF, Calcein/PI Cell Viability/Cytotoxicity Assay Kit, and BCA protein assay kit were purchased from Beyotime. Mouse IFN-β ELISA kit was purchased from Solarbio Life Sciences. Anti-HMGB1 antibody, anti-Calreticulin antibody, anti-gamma H2A.X (phospho S139) antibody, anti-Caspase-3 antibodywere ordered from Abcam. Cleaved Caspase-3 (Asp175) Antibody was ordered from Cell Signaling Technology (CST). DFNA5/GSDME Antibody - N-terminal was purchased from Affinity Biosciences. FITC-CD11c monoclonal antibody (N418), PE-CD80 (B7-1) monoclonal antibody (16-10A1), PE-Cyanine5-CD86 (B7-2) monoclonal antibody (GL1), FITC-CD3 monoclonal antibody (17A2), PE-CD4 monoclonal antibody (GK1.5), PE-Cyanine5-CD8a monoclonal antibody (53-6.7), FITC-F4/80 monoclonal antibody (BM8), PE-CD206 (NMR) monoclonal antibody (MR6F3) were purchased from eBioscience. Roswell Park Memorial Institute (RPMI) 1640 medium was purchased from Gibco. Fetal Bovine Serum (FBS) was purchased from Tianhang Biotechnology Co., Ltd.

Characterization Transmission electron microscopy (TEM) images were obtained by using a JEOL JEM-2100F transmission electron microscope. Dynamic light scattering (DLS) was measured by using Malvern Zetasizer Nano ZS. Ultraviolet-visible-near infrared region (UV-vis-NIR) absorption spectra were measured by using a Shimadzu 2600 UV-vis-NIR spectrophotometer. FTIR spectra were obtained by Brucker IFS80V FTIR spectrometer. The confocal laser scanning microscopy (CLSM) images were obtained by using the laser scanning confocal microscope Nikon AX or OLYMPUS BX81 (FluoView FV1000). High performance liquid chromatography (HPLC) was performed by iChrom 5100 (Elite). Fe content was detected by inductively coupled plasma atomic emission spectrometer (ICP-AES) measurements with a PerkinElmer Optima 3300DV.

Preparation of formulations The synthesis method of polyphenols and phosphate/phosphonate drugs mentioned in the universal study is similar to the above method. And it is important to note that Fe-apigenin and Fe-3’-hydroxyflavone cannot be collected by centrifugation.

0.6 mL of $FeCl_3 \cdot 6H_2O$ solution (20 mg/mL, H_2O) was added to 30 mL of deionized water, and 0.6 mL of galangin (5 mg/mL, DMSO) was added with magnetic stirring at 600 rpm. Subsequently, 50 μL 2’,3’-cGAMP (1 mg/mL) aqueous solution and 5.1 mL of zoledronic acid aqueous solution (2 mg/mL) was added. The mixture was centrifuged

at 8800 rpm for 10 min to collect the supernatant and the precipitate was re-dispersed in 1 mL of deionized water. This centrifugation step needs to be repeated for 2 times. The final solution is Fe-Gal@Fe-Zol-cGAMP nanodrug aqueous solution (4 mg/mL, zoledronic acid/galangin charge molar ratio = 3.25).

1 mL of Fe-Gal@Fe-Zol-cGAMP aqueous solution (4 mg/mL) was added to 4 mL of DOX aqueous solution (1 mg/mL) at 300 rpm stirring. After stirring for 24 h, the mixture was centrifuged at 5000 rpm for 10 min to collect the precipitate, and the supernatant was centrifuged at 8800 rpm for 10 min. The precipitate was collected and fixed to a volume of 1 mL. The final solution is DOX@Fe-Gal@Fe-Zol-cGAMP nanodrug aqueous solution (4mg/mL).

Response to GSH 50 μL of Fe-Gal@Fe-Zol-cGAMP was added into 900 μL of deionized water, then 50 μL of GSH aqueous solution (200 mM) was added. The mixture was then incubated in a 37 ℃ water bath for 24 h. After centrifugation, 100 ul of supernatant was added to 900 μL of water, followed by 20 μL of H_2O_2 aqueous solution (10 mM) and 30 μL of MB aqueous solution (1 mg/mL). The UV-vis absorption spectrum of the solution was monitored at different time points. H_2O_2 and Disodium terephthalate (2 mM) are used to detect ·OH generation by fluorescence spectrometer. To investigate release behavior of DOX from DOX@Fe-Gal@Fe-Zol-cGAMP, A dialysis bag (molecular weight cutoff of 1000 Da) containing 0.5 mL of DOX@Fe-Gal@Fe-Zol-cGAMP solution was incubated in 60 mL of deionized water or GSH solution (10 mM) at 37 ℃. 1 mL of release solution was extracted and an equal amount of fresh solution was added at different timepoints. Quantification of released DOX was performed by fluorescence spectrum.

Detection of intracellular Fe^{2+} 4T1 cells were seeded in a confocal dish at a density of 1×10^5 cells per well. After incubation with Fe-Gal, Fe-Gal@Fe-cGAMP, Fe-Gal@Fe-Zol, Fe-Gal@Fe-Zol-cGAMP, and DOX@Fe-Gal@Fe-Zol-cGAMP for 24 h, 4T1 cells were incubated with FerroOrange probe for 30 min and observed using CLSM.

Live/dead cells detection 4T1 cells were seeded in a confocal dish as aforesaid. And then 4T1 cells were incubated with Fe-Gal, Fe-Gal@Fe-cGAMP, Fe-Gal@Fe-Zol, Fe-Gal@Fe-Zol-cGAMP, and DOX@Fe-Gal@Fe-Zol-cGAMP for 24 h. An appropriate amount of calcein AM/ PI working solution was added. The cells were incubated at 37 ºC for 30 min. Subsequently, the 4T1 cells were examined by CLSM (Calcein AM, Ex/Em = 488/517 nm; PI-DNA, Ex/Em = 543/617 nm).

Detection of intracellular ROS level 4T1 cells were seeded in the confocal dish as aforesaid. After incubation with Fe-Gal, Fe-Gal@Fe-cGAMP, Fe-Gal@Fe-Zol, Fe-Gal@Fe-Zol-cGAMP, and DOX@Fe-Gal@Fe-Zol-cGAMP for 24 h, the medium was removed and the cells were washed by 1640 medium for 3 times. Then the cells were incubated with DCFH-DA probe for 30 min and observed using CLSM.

DNA damage 4T1 cells were cultured in the confocal dish as aforesaid. After attachment, the cells were incubated with Fe-Gal, Fe-Gal@Fe-cGAMP, Fe-Gal@Fe-Zol, Fe-Gal@Fe-Zol-cGAMP, and DOX@Fe-Gal@Fe-Zol-cGAMP for 24 h. Then the cells were successively fixed with 4% paraformaldehyde, permeabilized with 0.1% Triton X-100 for 10 min. After washing for 3 times, the cells were blocked and incubated with the anti-γ-H2AX antibody overnight at 4 ℃, then the cells were treated

with Goat Anti-Rabbit IgG H&L/FITC antibody for 1 h in dark and Hoechst 33342 for 15 min, respectively. All the samples were observed using CLSM.

LDH detection 4T1 cells were seeded in a 24-well plate at a density of 5×10^4 cells per well. After attachment, the cells were incubated with Fe-Gal, Fe-Gal@Fe-cGAMP, Fe-Gal@Fe-Zol, Fe-Gal@Fe-Zol-cGAMP, and DOX@Fe-Gal@Fe-Zol-cGAMP for 24 h. One hour before the end of treatment, the LDH release reagent provided in the kit was added to the blank group (only cells) as "High Control”. The culture medium was centrifuged at 500×g for 5 min. 120 μL supernatant from each well was transferred into a new 96-well plate. 60 μL LDH working solution was added per well and the mixture was incubated at room temperature in the dark for 30 min. The absorbance of all samples was measured with a plate reader at 490 nm.

Western Blot Assay 4T1 cells after different treatments were washed 3 times by pre-chilled PBS and lysed on ice. The supernatant was obtained after centrifugation (12000 rpm, 10 min) at 4 ℃. Protein concentration was measured by a BCA protein kit (Biotechnology, China). Loading buffer was added, and the mixture was boiled at 100 °C for 15 min to obtain denatured protein. After electrophoresis, transmembrane, and blocking, the primary antibody was added and incubated with polyvinylidene fluoride membranes for 12 h at 4 °C. The next day, the horseradish peroxidase-conjugated secondary antibody was incubated at room temperature for 30 min and the mixed enhanced chemiluminescent substrate was added, then electrochemiluminescence bands were obtained by a chemiluminescent imaging system.

Cytotoxicity assay For the cytotoxicity assay of DOX@Fe-Gal@Fe-Zol-cGAMP toward 4T1 cells with different inhibitors, 4T1 cells were seeded in 96-well culture plates as aforesaid. After incubation for 24 h, Ac-DEVD-CHO (2×10^{-5} mol L^{-1}), 2-Bromohexadecanoic acid (1×10^{-4} mol L^{-1}) were added into 96-well culture plates. After incubation for 1 h, DOX@Fe-Gal@Fe-Zol-cGAMP (50 μg mL^{-1}) was added into 96-well culture plates. After incubation, 10 μL of CCK-8 was added and the cells were incubated for another 1.5 h at 37 ℃. The OD value of each well was measured by microplate reader at 450 nm.

ICD effects *in vitro* To examine the ICD effect of DOX@Fe-Gal@Fe-Zol-cGAMP, the release of DAMPs was examined *in vitro*, which included exposure of calreticulin (CRT), release of HMGB1, and secretion of ATP. 4T1 cells were cultured in the confocal dish as aforesaid. The cells were incubated with Fe-Gal, Fe-Gal@Fe-cGAMP, Fe-Gal@Fe-Zol, Fe-Gal@Fe-Zol-cGAMP, and DOX@Fe-Gal@Fe-Zol-cGAMP for 24 h. After washing by PBS for 3 times, the cells were sequentially fixed with 4% paraformaldehyde and permeabilized with 0.1% Triton X-100 for 10 min. After washing, the cells were blocked and incubated with the anti-HMGB1 or anti-CRT antibody overnight at 4 ℃, then the cells were incubated with Goat Anti-Rabbit IgG H&L/FITC antibody for 2 h and Hoechst 33342 for 15 min, respectively. All the samples were observed using CLSM. For quantification of released HMGB1 in culture medium, the medium of 4T1 cells after different treatments were collected. The released HMGB1 content was measured with a Mouse HMGB1 Elisa Kit according to vendors’ instructions.

The secretion of ATP was detected using an ATP assay kit. 4T1 cells were seeded in a 6-well culture plate at a density of 4×10^5 cells per well and incubated overnight. And then, cells were treated with Fe-Gal, Fe-Gal@Fe-cGAMP, Fe-Gal@Fe-Zol, Fe-Gal@Fe-Zol-cGAMP, and DOX@Fe-Gal@Fe-Zol-cGAMP for 24 h, respectively. After incubation, 100 μL of ATP assay working solution was added into a black 96-well cell culture plate, and then 20 μL of the supernatant was added. Measure the RLU with a luminometer (integration time: 1000 ms).

***In vitro* DC maturation, macrophage polarization, and pro-inflammatory cytokine secretion** Murine bone marrow cells were isolated from BALB/c mice as reported previously, and bone marrow-derived macrophages (BMDMs) and bone marrow-derived dendritic cells (BMDCs) are generated by GM-CSF-induced differentiation. For BMDC maturation experiments, 4T1 cells were seeded in 6-well plates overnight. Then the medium was replaced by the medium contained Fe-Gal, Fe-Gal@Fe-cGAMP, Fe-Gal@Fe-Zol, Fe-Gal@Fe-Zol-cGAMP, and DOX@Fe-Gal@Fe-Zol-cGAMP, respectively. After incubation for 6 h, the medium was replaced by fresh medium. After incubation for another 18 h, IFN-β content in the culture medium was measured with a Mouse IFN-β Elisa Kit according to vendors' instructions. The supernatant of tumor cells was collected and added into BMDCs suspension at a ratio of 1:1. After 24 h incubation, IFN-β, IFN-γ, and TNF-α content in culture medium was measured as aforesaid. The non-adherent cells were harvested, washed by hanks' balanced salt solution (HBSS) and incubated with anti-CD11c-FITC, anti-CD80-PE, and anti-CD86-PE-Cy5 monoclonal antibodies for 30 min. After washing by HBSS, the cells were analyzed by flow cytometry.

For BMDMs polarization study, the adherent cells were harvested and seeded on a 6-well plate. Similarly, after attachment, the BMDMs were incubated with the supernatant of tumor cells for 24 h. After 24 h incubation, the cells were collected, washed by HBSS and incubated with anti-CD11b-PE, anti-F4/80-FITC, and anti-CD86-PE-Cy5 monoclonal antibodies for 30 min. After washing by HBSS, the cells were analyzed by flow cytometry.

Molecular dynamic simulations. The molecular dynamic (MD) simulations were carried out with the Lammps using the pcff force field. Prior to MD simulations, all of the molecules were optimized by b3lyp/6-31g(d,p) with the Gaussian09 program. For Fe-galangin, the MD simulations were performed in a 10×10×10 nm^3 box, where the 60 Fe^{3+} and 180 galangin molecules were placed uniformly. The total atoms of the system were 9606. The initial energy minimization was performed using GC. After that, with the time step of 1 fs, the system ran for 10 ns at 300 K and 1 atm in the control of Berendsen's barostat with an isothermal compressibility constant of 4.5×10^{-5} was used to control the pressure at 1.01325×10^5 Pa, respectively. For Fe-galangin@Fe-zoledronic acid, 60 Fe^{3+} and 280 galangin or 12 zoledronic acid dimers were placed in the above-mentioned box. The Gibbs free energy was calculated by the Gaussian09 program, we calculated the energy of the reactor and productor, Gibbs free energy is the difference in energy before and after a reaction.

***In vivo* biodistribution of ^{ICG}Fe-Gal@Fe-Zol** 1×10^6 4T1 cells were subcutaneously injected into the right mammary fat pads of BALB/c mice to establish the orthotopic

4T1 breast tumor model. When the tumors reached ~400 mm^3, mice were intravenously injected with ICG and ^{ICG}Fe-Gal@Fe-Zol (5 mg/kg). Whereafter, the mice were anesthetized by isoflurane and imaged in a small animal imaging system at 0.5, 1, 2.5, 9, 11, 24, and 48 h post-injection. The excitation wavelength was 808 nm. The heart, liver, spleen, lung, kidney, and tumor were collected and imaged. In addition, three mice in each group were collected blood from the orbital venous plexus at 0.5, 2, 3.5, 5.5, and 8 h post-injection, and fluorescence intensity was measured by imaging.

***In vivo* antitumor** Orthotopic 4T1 breast tumor model was constructed as aforesaid. When the tumors reached a volume of ~100 mm^3, mice were intravenously injected with Fe-Gal, Fe-Gal@Fe-cGAMP, Fe-Gal@Fe-Zol, Fe-Gal@Fe-Zol-cGAMP, and DOX@Fe-Gal@Fe-Zol-cGAMP (10 mg/kg) at day 0. Tumor volumes and body weights were recorded every other day up to 14 days. The tumor volume was calculated according to the following equation:

$$\text{tumor volume} = \text{length}\times\text{width}^2\times 0.5\ \text{mm}^3.$$

After treatments, the main organs including the heart, liver, spleen, lungs, kidneys, and tumor were collected for H&E staining analysis. Blood was used for liver and kidney function analysis.

Immune response analysis Following the aforementioned Orthotopic 4T1 breast tumor model, the tumor-draining lymph nodes (TDLNs) were collected for DCs maturation analysis, The TDLNs were ground into a single cell suspension and filtered through a sieve. Then the cells were blocked and incubated with anti-CD11c-FITC, anti-CD80-PE, and anti-CD86-PE-Cy5 monoclonal antibodies for 30 min. The cells are collected by centrifugation for flow cytometry.

Following the aforementioned Orthotopic 4T1 breast tumor model, the tumors were collected for immune response and TAM polarization analysis. The tumors were ground into a single cell suspension and filtered through a sieve. For TAM polarization analysis, the cells were stained with anti-F4/80-FITC, anti-CD206-PE, and anti-CD86-PE-Cy5 monoclonal antibodies for 30 min. For cytotoxic T lymphocyte cells (CTLs) and T helper cells (Ths) infiltration analysis, the cells were stained with anti-CD3-FITC, anti-CD4-PE, and anti-CD8a-PE-Cy5 monoclonal antibodies for 30 min. Then the cells were centrifuged and analyzed using flow cytometry.

The tumor section was stained with anti-CD4 and anti-CD8a for IHC analysis, respectively.

***In vivo* metastasis inhibition** Following the aforementioned Orthotopic 4T1 breast tumor model, lung tissues were isolated from mice at 30 days post-treatment. After photography, lungs were used for H&E staining analysis.

***In vivo* murine model of 4T1-based breast cancer metastasis to bone** 25 μL of 4T1 breast cancer cells (density of 4×10^7 cells/mL) in PBS were directly injected into the tibiae plateau (right leg) of female BALB/c mice via a percutaneous approach. Three days later, mice were randomly divided into 6 groups (n = 3 per group) and intravenously injected with Fe-Gal, Fe-Gal@Fe-cGAMP, Fe-Gal@Fe-Zol, Fe-Gal@Fe-Zol-cGAMP, and DOX@Fe-Gal@Fe-Zol-cGAMP (10 mg/kg). The circumference of the tumor-inoculated leg of the mouse was recorded every other day until the tenth day. The right leg tibiae of mice after various treatments were scanned

by micro-computed tomography (micro-CT). After 3D reconstruction, the bone volume (BV), bone volume/total volume (BV/TV), bone surface (BS), and bone mineral density (BMD) of the tibiae were measured.

Figure S1. (a) TEM images and (b) UV-vis absorption spectra of Fe-3’,4’-dihydroxyflavone and Fe-3’,4’-dihydroxyflavone@Fe-zoledronic acid. (c) TEM images and (d) UV-vis absorption spectra of Fe-3’-hydroxyflavone and Fe-3’-hydroxyflavone@Fe-zoledronic acid. (e) TEM images and (f) UV-vis absorption spectra of Fe-5’-hydroxyflavone and Fe-5’-hydroxyflavone@Fe-zoledronic acid. All scale bars are 100 nm.

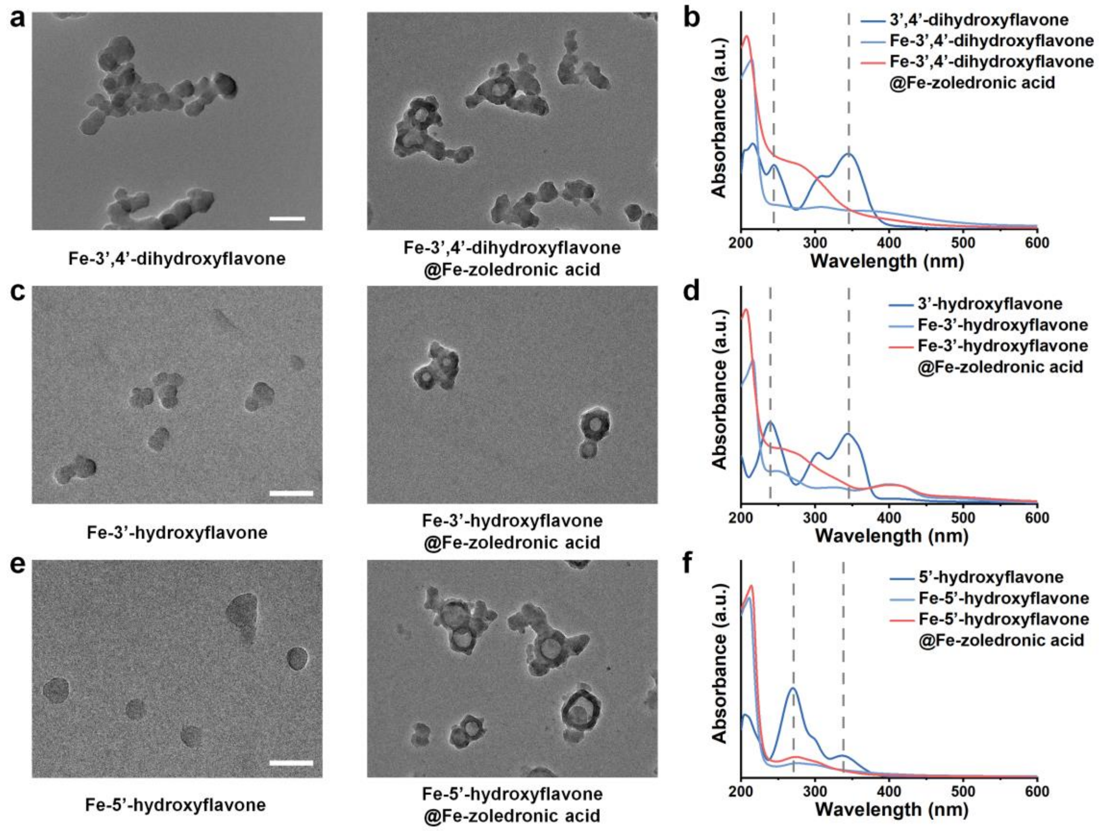

Figure S2. UV-vis absorption spectra of (a) Fe-zoledronic acid, (b) Fe-ATP, (c) Fe-ADP, (d) Fe-AMP.

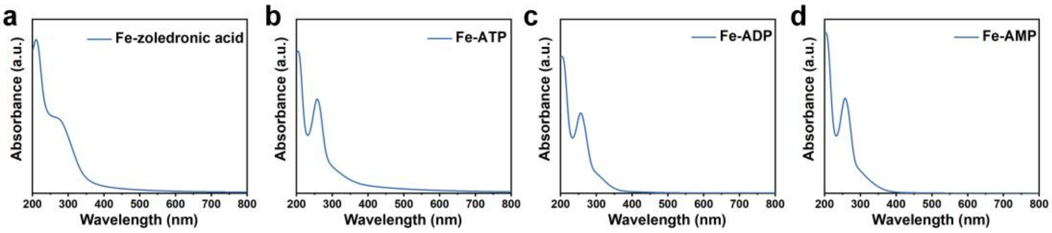

Figure S3. UV-vis absorption spectra of a series of polyphenols (galangin, myricetin, quercetin, baicalein, luteolin, fisetin, apigenin, EGCG, shikonin), Fe-polyphenols, Fe-polyphenols@Fe-zoledronic acid, Fe-polyphenols@Fe-ATP, Fe-polyphenols@Fe-ADP, Fe-polyphenols@Fe-AMP.

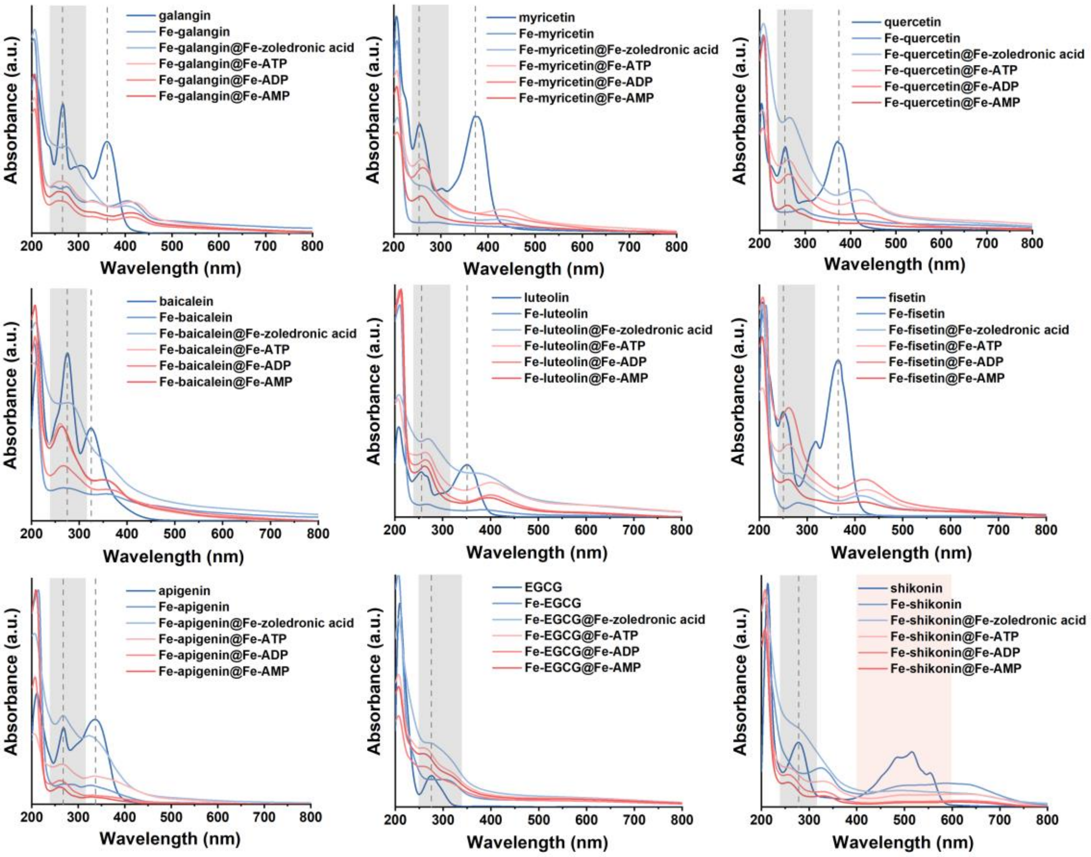

Figure S4. TEM images of a series of polyphenols (galangin, myricetin, quercetin, baicalein, luteolin, fisetin, apigenin, EGCG, shikonin), Fe-polyphenols, Fe-polyphenols@Fe-zoledronic acid, Fe-polyphenols@Fe-ATP, Fe-polyphenols@Fe-ADP, Fe-polyphenols@Fe-AMP. All scale bars are 50 nm.

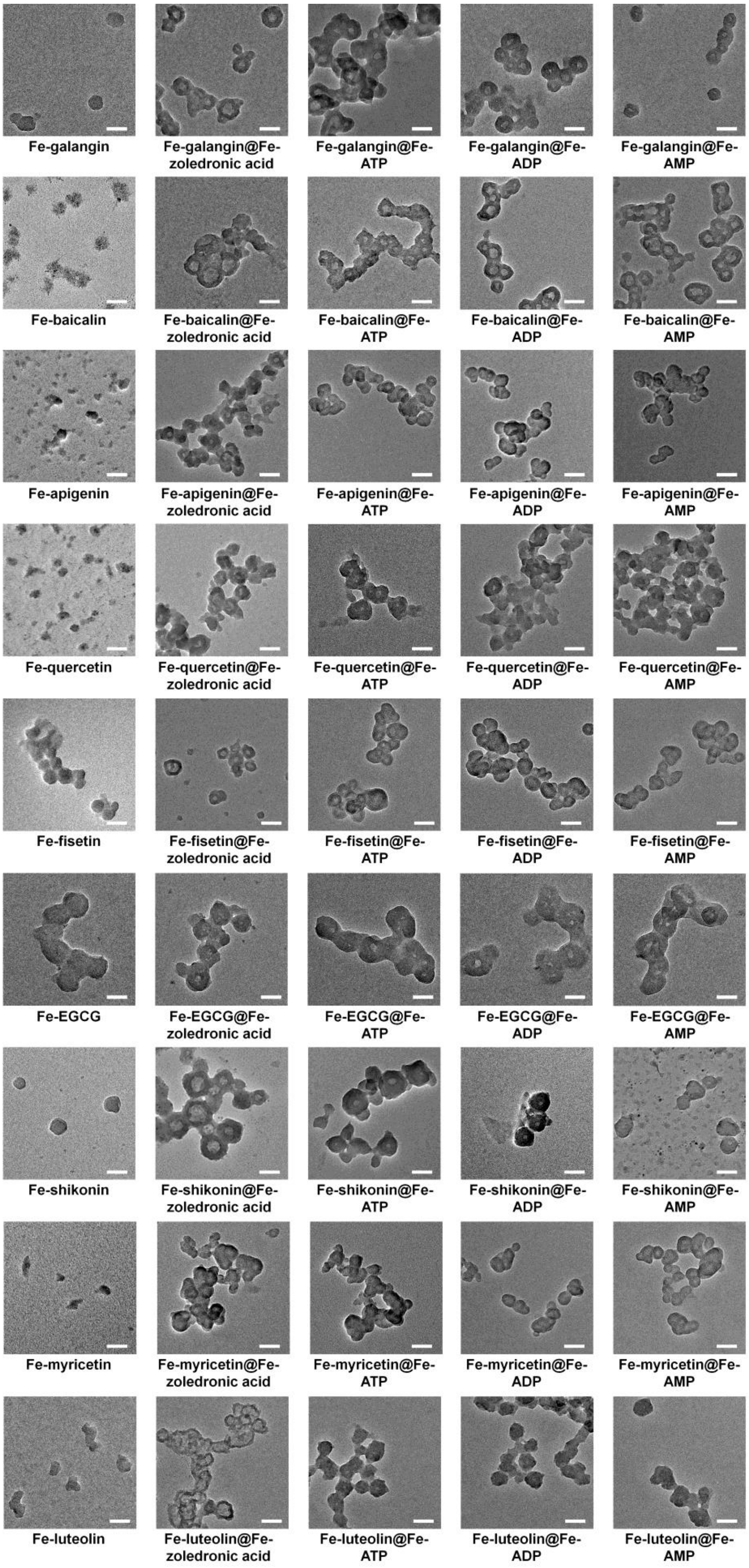
Fe-galangin
Fe-galangin@Fe-zoledronic acid
Fe-galangin@Fe-ATP
Fe-galangin@Fe-ADP
Fe-galangin@Fe-AMP
Fe-baicalin
Fe-baicalin@Fe-zoledronic acid
Fe-baicalin@Fe-ATP
Fe-baicalin@Fe-ADP
Fe-baicalin@Fe-AMP
Fe-apigenin
Fe-apigenin@Fe-zoledronic acid
Fe-apigenin@Fe-ATP
Fe-apigenin@Fe-ADP
Fe-apigenin@Fe-AMP
Fe-quercetin
Fe-quercetin@Fe-zoledronic acid
Fe-quercetin@Fe-ATP
Fe-quercetin@Fe-ADP
Fe-quercetin@Fe-AMP
Fe-fisetin
Fe-fisetin@Fe-zoledronic acid
Fe-fisetin@Fe-ATP
Fe-fisetin@Fe-ADP
Fe-fisetin@Fe-AMP
Fe-EGCG
Fe-EGCG@Fe-zoledronic acid
Fe-EGCG@Fe-ATP
Fe-EGCG@Fe-ADP
Fe-EGCG@Fe-AMP
Fe-shikonin
Fe-shikonin@Fe-zoledronic acid
Fe-shikonin@Fe-ATP
Fe-shikonin@Fe-ADP
Fe-shikonin@Fe-AMP
Fe-myricetin
Fe-myricetin@Fe-zoledronic acid
Fe-myricetin@Fe-ATP
Fe-myricetin@Fe-ADP
Fe-myricetin@Fe-AMP
Fe-luteolin
Fe-luteolin@Fe-zoledronic acid
Fe-luteolin@Fe-ATP
Fe-luteolin@Fe-ADP
Fe-luteolin@Fe-AMP

Figure S5. SEM image of Fe-Gal@Fe-Zol. Scale bar is 100 nm.

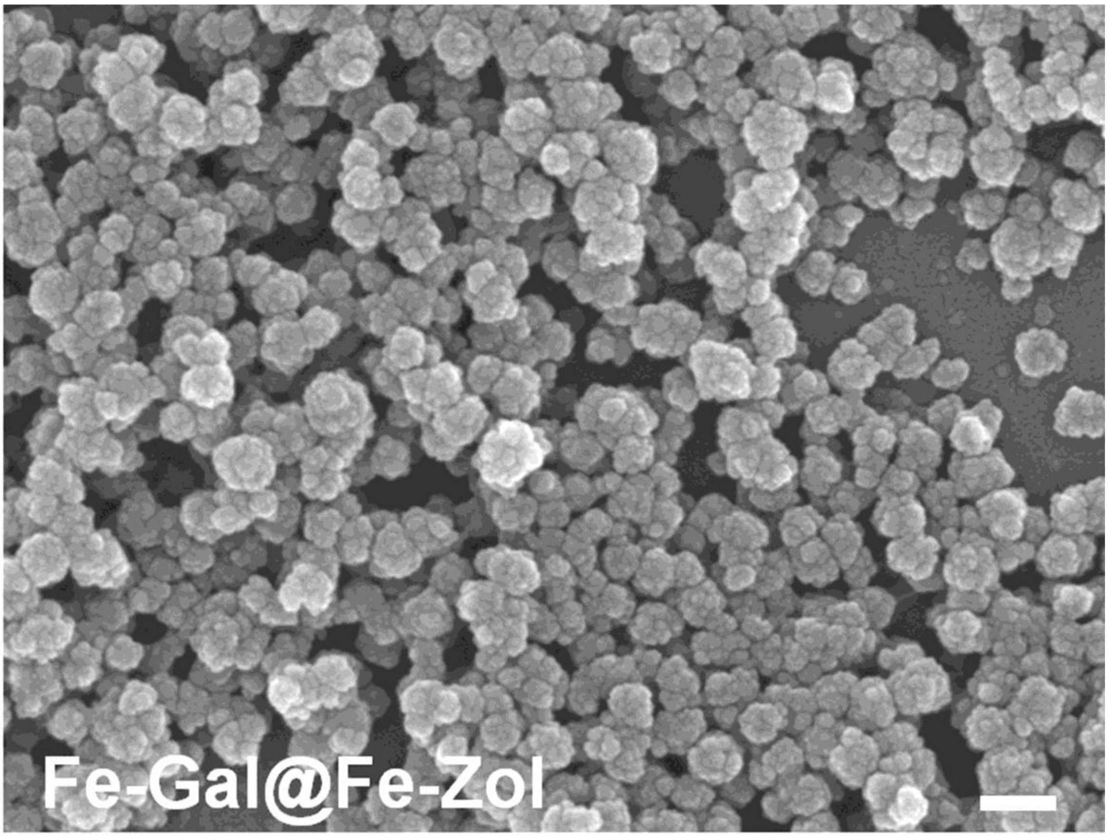

Figure S6. EDS spectrum of Fe-Gal@Fe-Zol.

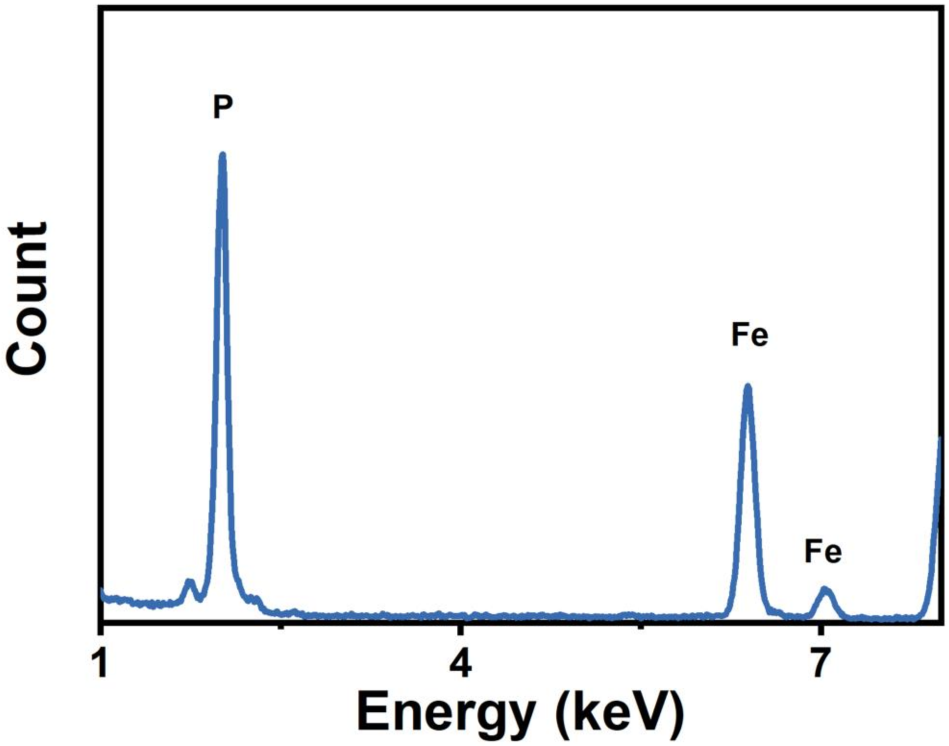

Figure S7. (a) TEM images of Fe-Gal@Fe-Zol at different zoledronic/galangin charge ratios. Scale bar is 100 nm. (b) Cavity size of DOX@Fe-Gal@Fe-Zol-cGAMP at different zoledronic acid/galangin charge ratios (n =26). Data are shown as mean ± SD; n represents the number of biologically independent samples.

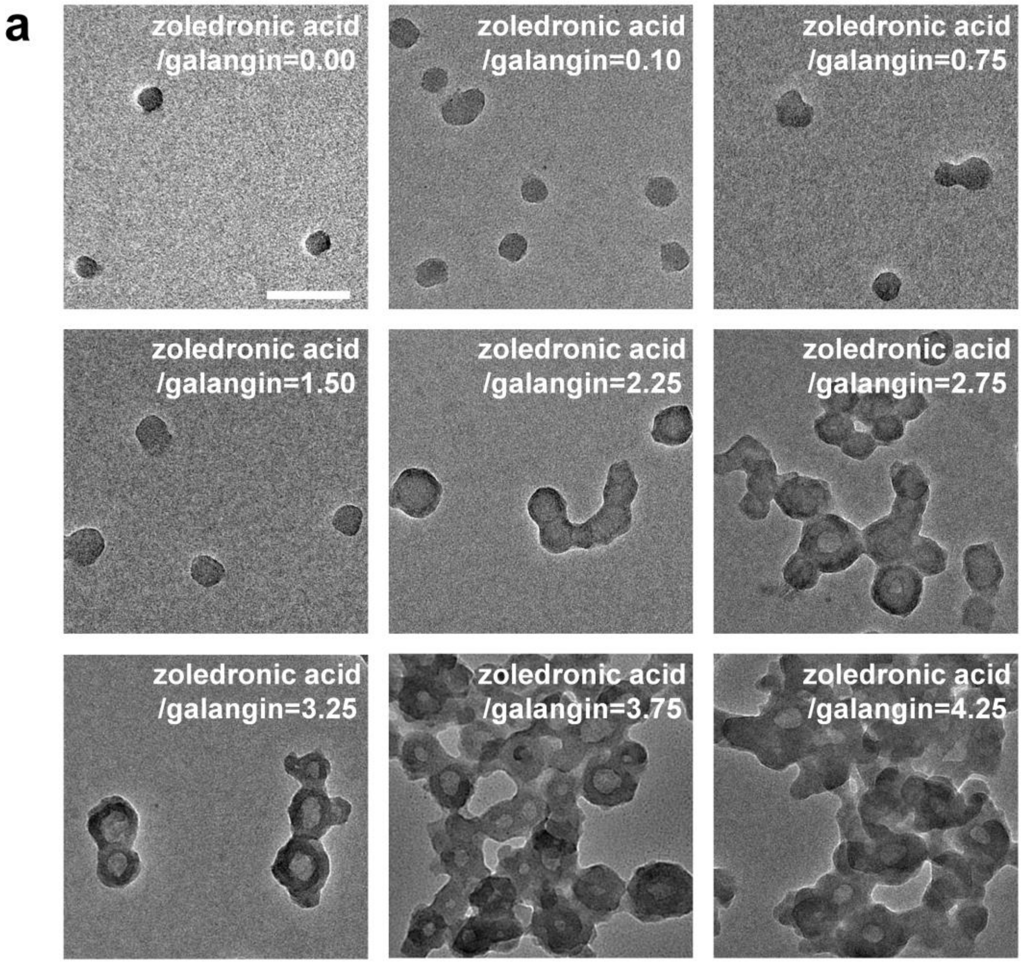

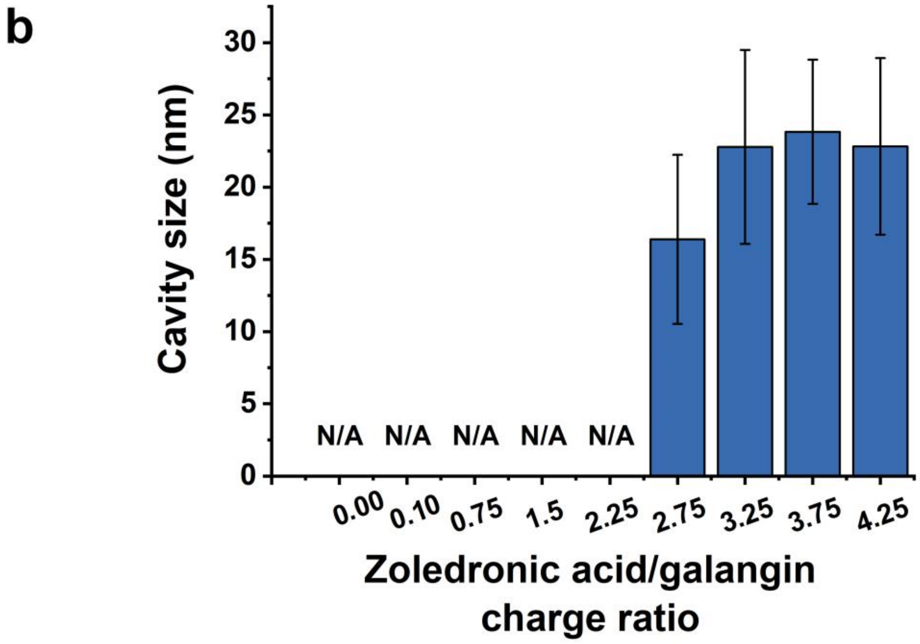

Figure S8. Cell viability of 4T1 cells under different treatments determined by CCK-8 assay (n=5). Data are shown as mean ± SD; n represents the number of biologically independent samples.

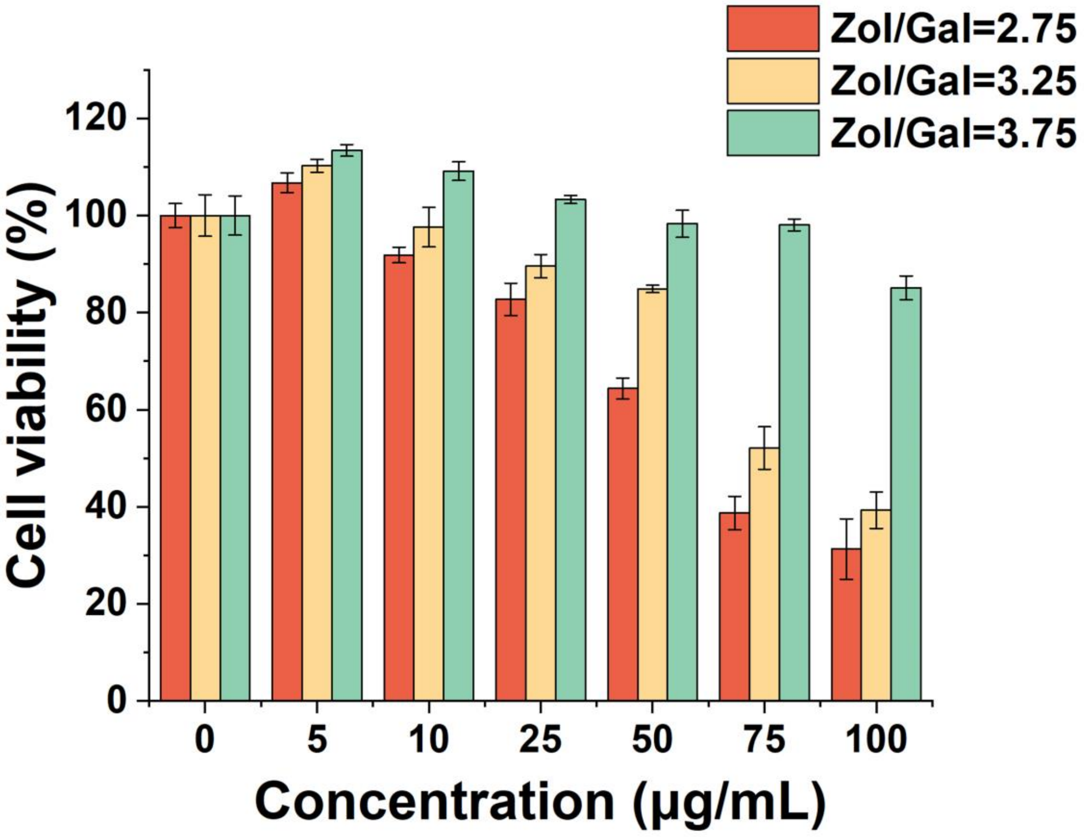

Figure S9. (a) TEM images, (b) UV-vis absorption spectra, and (c) FTIR spectra of DOX@Fe-Gal@Fe-Zol, DAC@Fe-Gal@Fe-Zol, ICG@Fe-Gal@Fe-Zol and RB@Fe-Gal@Fe-Zol. Scale bar is 100 nm.

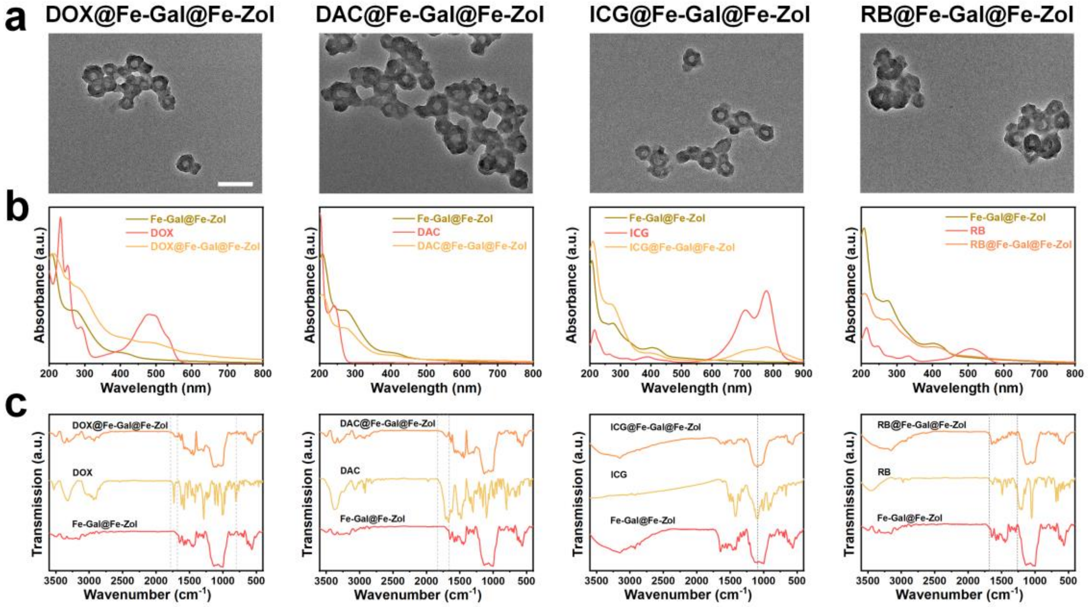

Figure S10. The Δ*f* traces of Fe-Gal from QCM upon the addition of cGAMP.

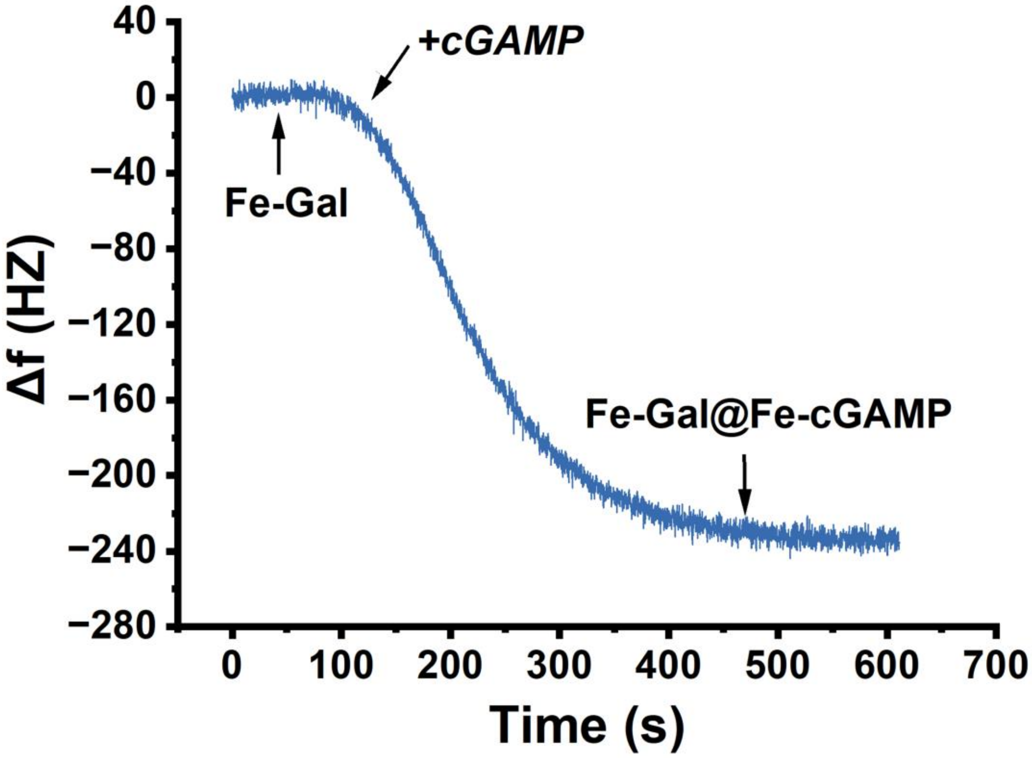

Figure S11. IFN-β secretion levels of L929 cells upon different incubation (n = 4). Group: (I) Control, (II)-(IV) DOX@Fe-Gal@Fe-Zol-cGAMP prepared when the feeding amount of cGAMP is 25, 50, and 100 μg, respectively. Data are shown as mean ± SD; n represents the number of biologically independent samples.

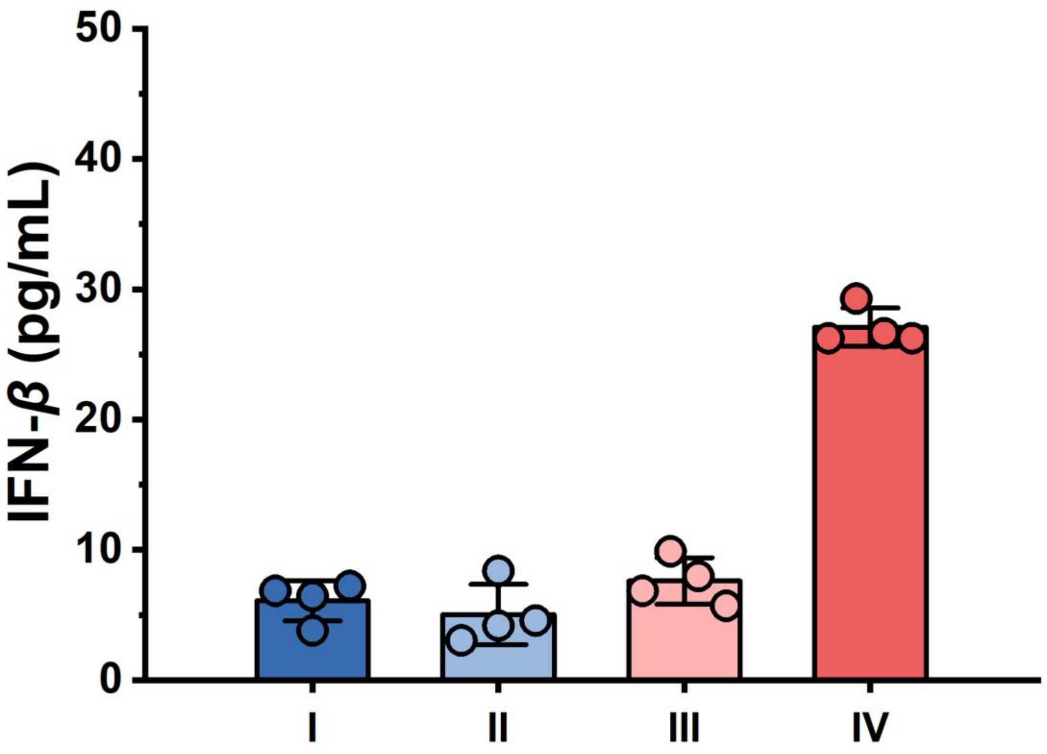

Figure S12. Photograph of Fe-Gal@Fe-Zol-cGAMP (left) and Fe-Gal@Fe-Zol-cGAMP incubated with GSH (10 mM) for 24 hours (right).

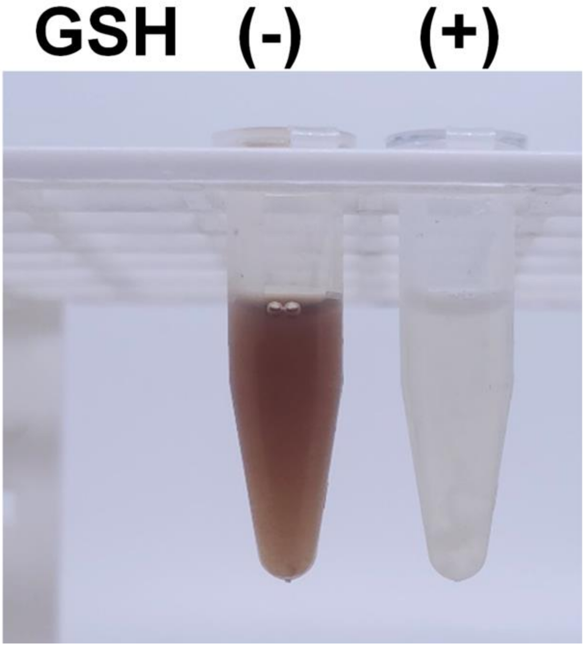

Figure S13. TEM images of (a) Fe-Gal@Fe-Zol-cGAMP, (b) Fe-Gal@Fe-Zol-cGAMP incubated with GSH (10 mM) for 12 hours and (c) Fe-Gal@Fe-Zol-cGAMP incubated with GSH (10 mM) for 24 hours. Scale bar is 100 nm.

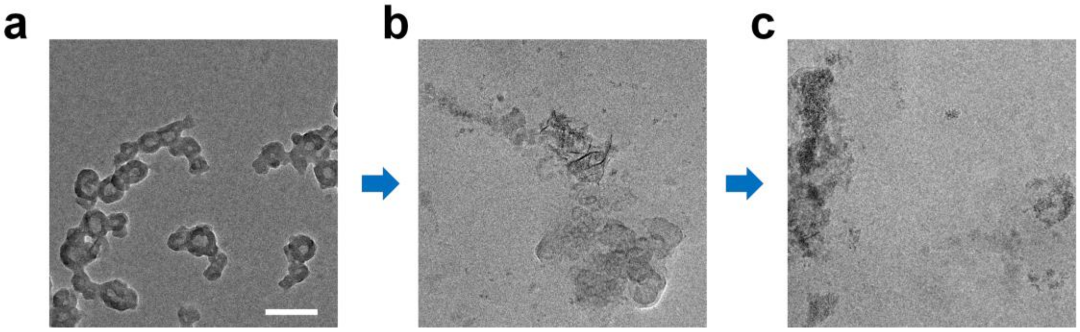

Figure S14. (a) Temporal evolution of UV-vis absorption spectra of MB solution in the presence of H_2O_2 and Fe-Gal@Fe-Zol-cGAMP incubated with GSH. (b) Fluorescence spectra of disodium terephthalate upon different treatments.

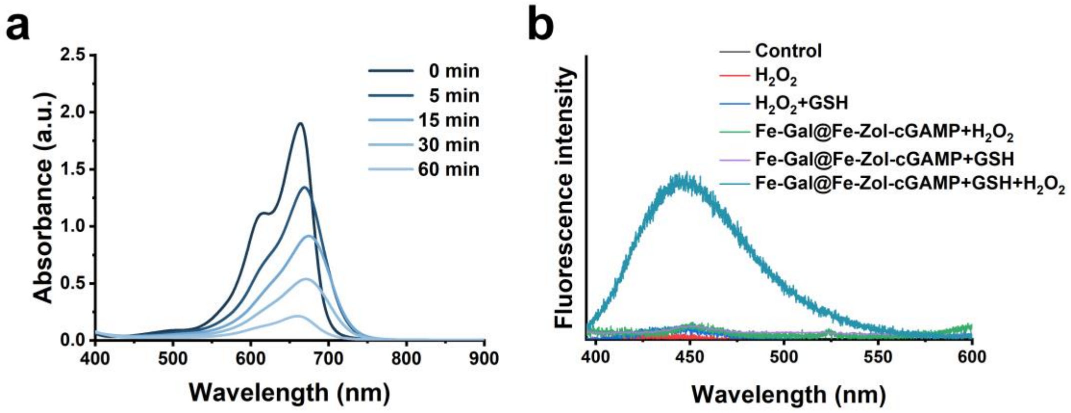

Figure S15. DOX release curves of DOX@Fe-Gal@Fe-Zol-cGAMP in the absence and presence of GSH (n = 3). Data are shown as mean ± SD; n represents the number of biologically independent samples.

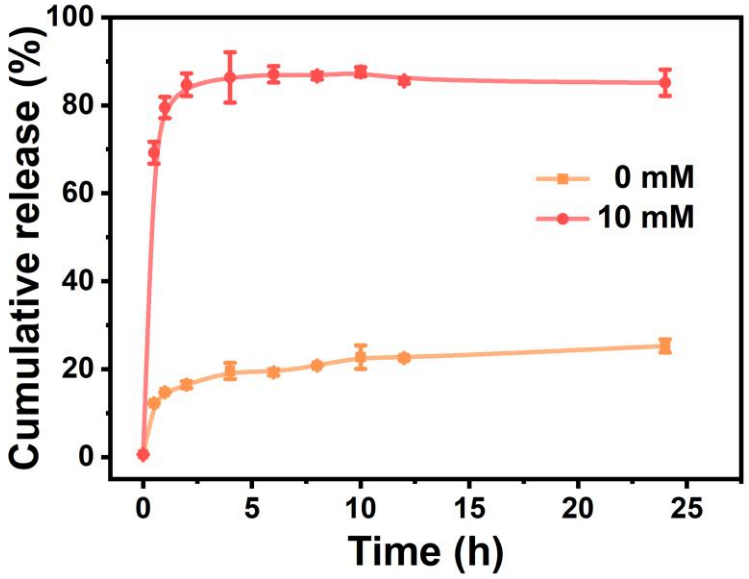

Figure S16. Concentration of adsorbed proteins on the surface of DOX@Fe-Gal@Fe-Zol-cGAMP (n = 3). Data are shown as mean ± SD; n represents the number of biologically independent samples.

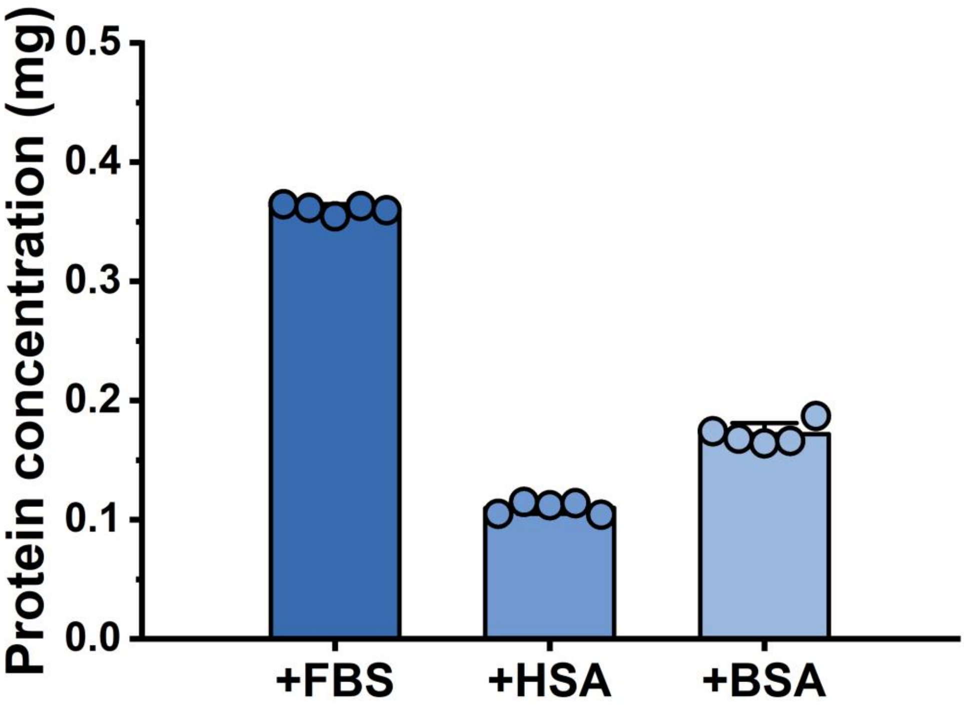

Figure S17. TEM images of (a) Fe-PEG-Dopamine and (b) Fe-PEG-Dopamine@Fe-Zol. Scale bar is 100 nm. (c) The Δ*f* traces of Fe-PEG-Dopamine from QCM upon the addition of zoledronic acid. Dopa refers to dopamine.

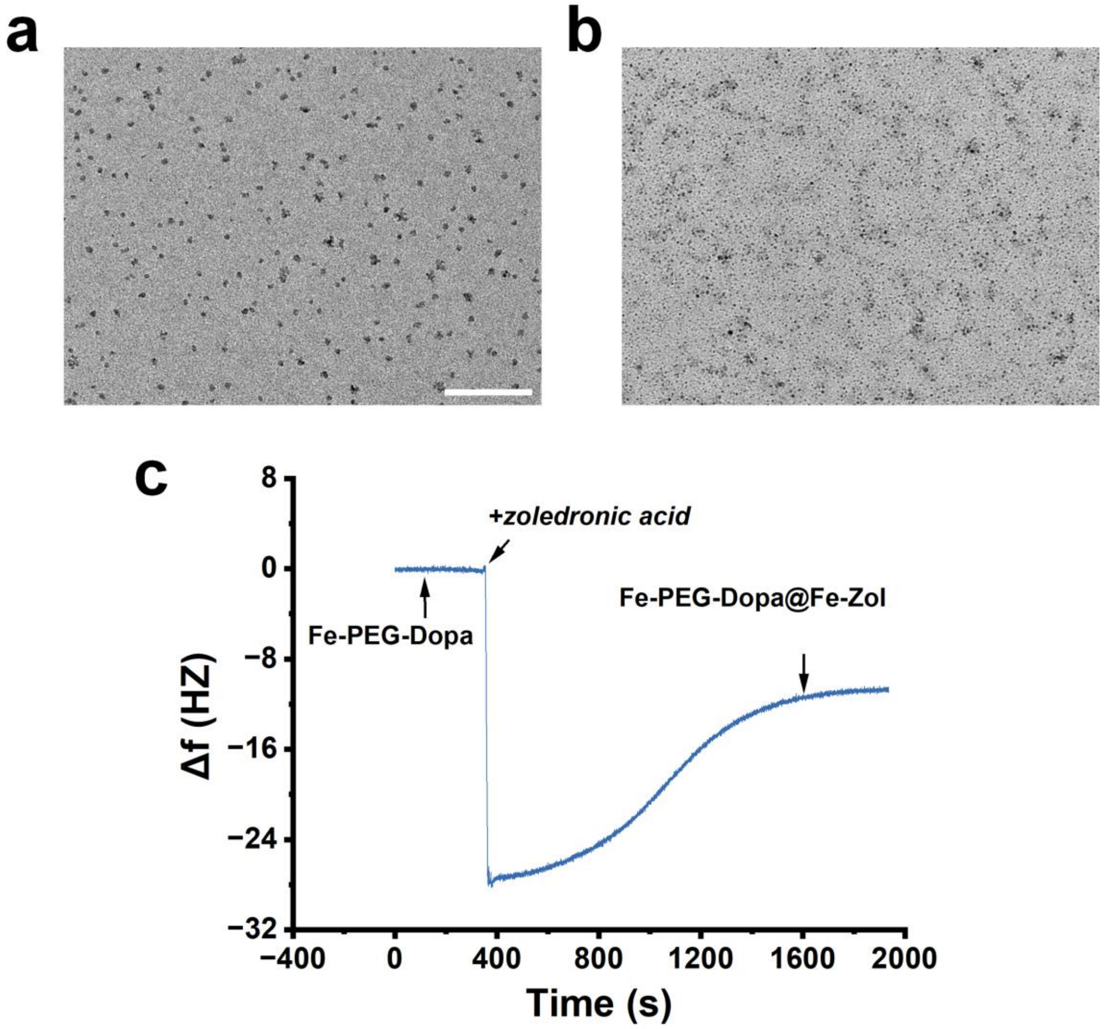

Figure S18. Cell viability of L929 and 4T1 cells under different treatments determined by CCK-8 assay (n=5). Data are shown as mean ± SD; n represents the number of biologically independent samples.

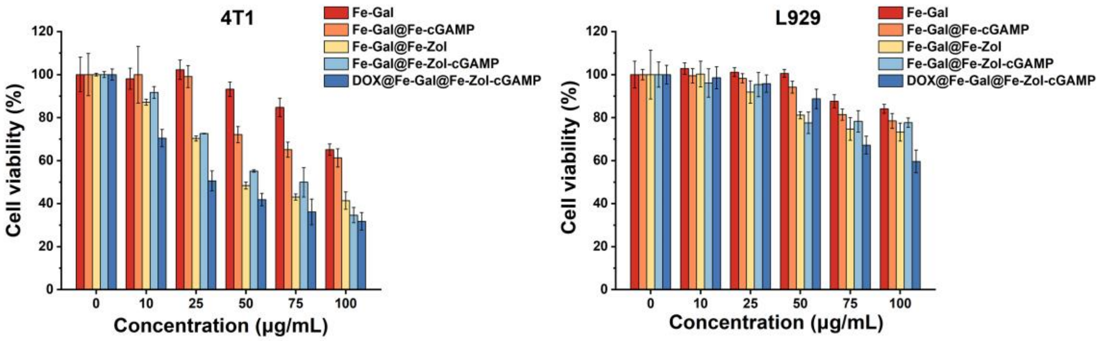

Figure S19. Live/dead cell staining images via Calcein AM/PI staining of 4T1 cells upon different treatments. Red fluorescence represents dead cells and the green fluorescence represents living cells. Scale bar is 100 μm. Group: (I) Control, (II) Fe-Gal, (III) Fe-Gal@Fe-cGAMP, (IV) Fe-Gal@Fe-Zol, (V) Fe-Gal@Fe-Zol-cGAMP, (VI) DOX@Fe-Gal@Fe-Zol-cGAMP.

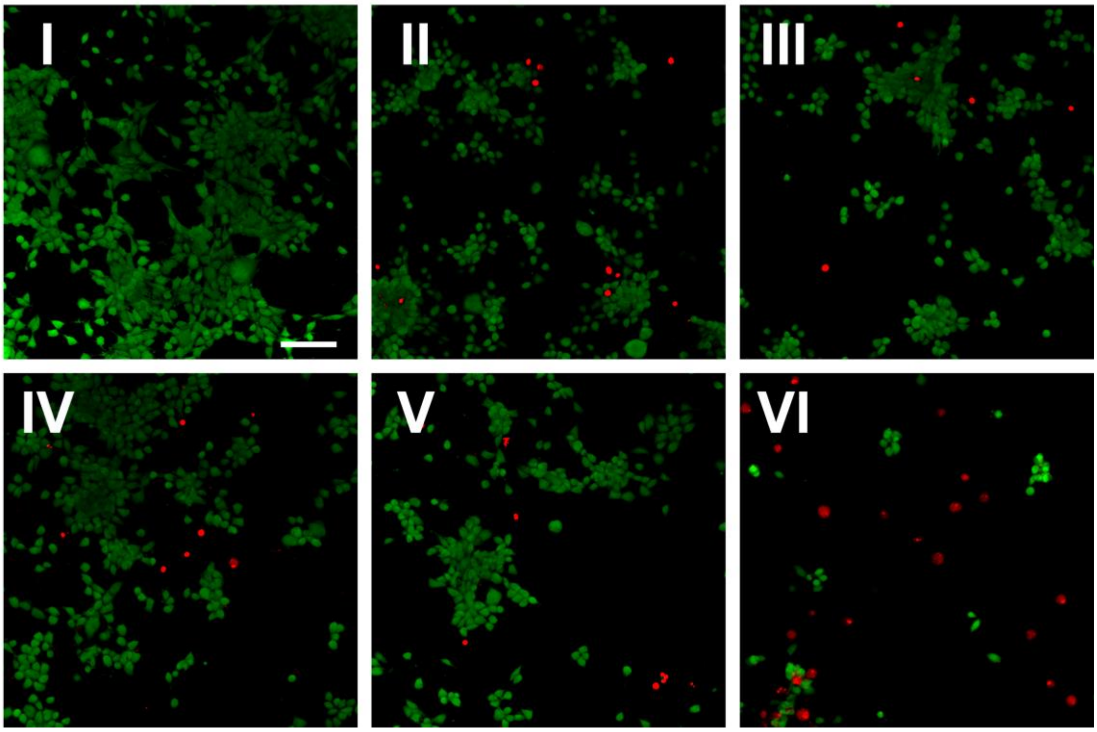

Figure S20. CLSM images of Fe^{2+} levels via FerroOrange staining in 4T1 cells upon different treatments. Scale bar is 50 μm. Group: (I) Control, (II) Fe-Gal, (III) Fe-Gal@Fe-cGAMP, (IV) Fe-Gal@Fe-Zol, (V) Fe-Gal@Fe-Zol-cGAMP, (VI) DOX@Fe-Gal@Fe-Zol-cGAMP.

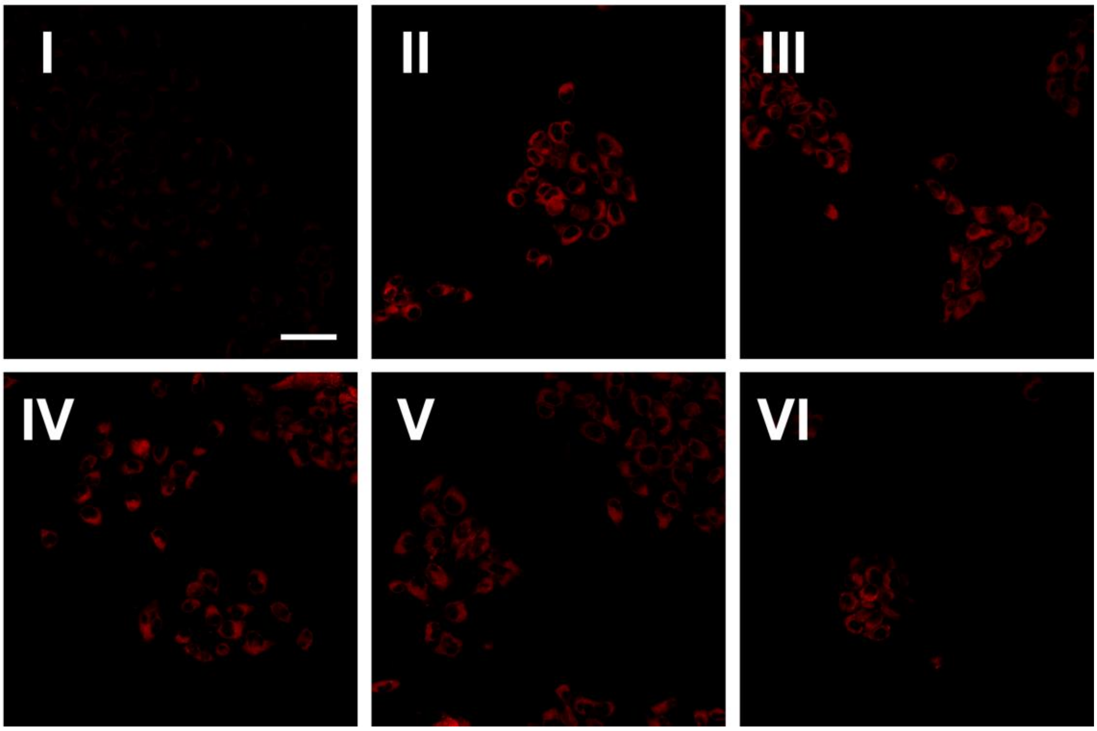

Figure S21. CLSM images of ROS levels via DCFH-DA staining in 4T1 cells upon different treatments. Scale bar is 50 μm. Group: (I) Control, (II) Fe-Gal, (III) Fe-Gal@Fe-cGAMP, (IV) Fe-Gal@Fe-Zol, (V) Fe-Gal@Fe-Zol-cGAMP, (VI) DOX@Fe-Gal@Fe-Zol-cGAMP.

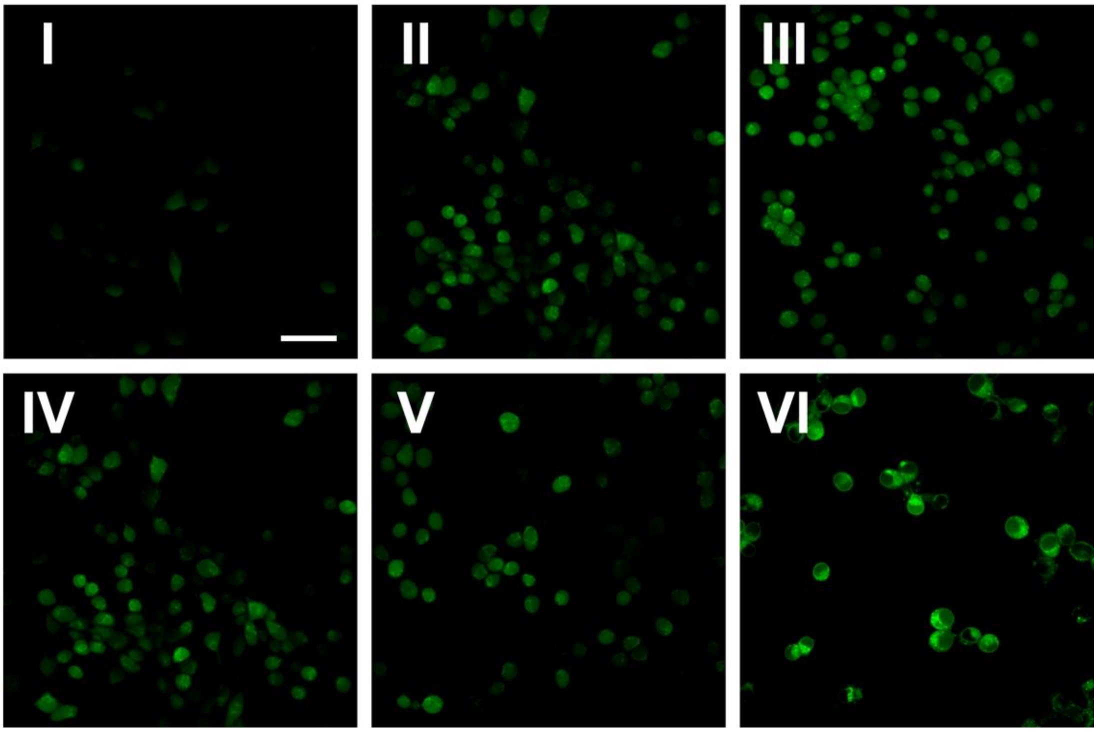

Figure S22. CLSM images and quantitative MFI of γ-H2AX in 4T1 cells upon different treatments (n = 3). Scale bar is 50 μm. Group: (I) Control, (II) Fe-Gal, (III) Fe-Gal@Fe-cGAMP, (IV) Fe-Gal@Fe-Zol, (V) Fe-Gal@Fe-Zol-cGAMP, (VI) DOX@Fe-Gal@Fe-Zol-cGAMP. Data are shown as mean ± SD; n represents the number of biologically independent samples. **P* < 0.05, ***P* < 0.01, and ****P* < 0.001.

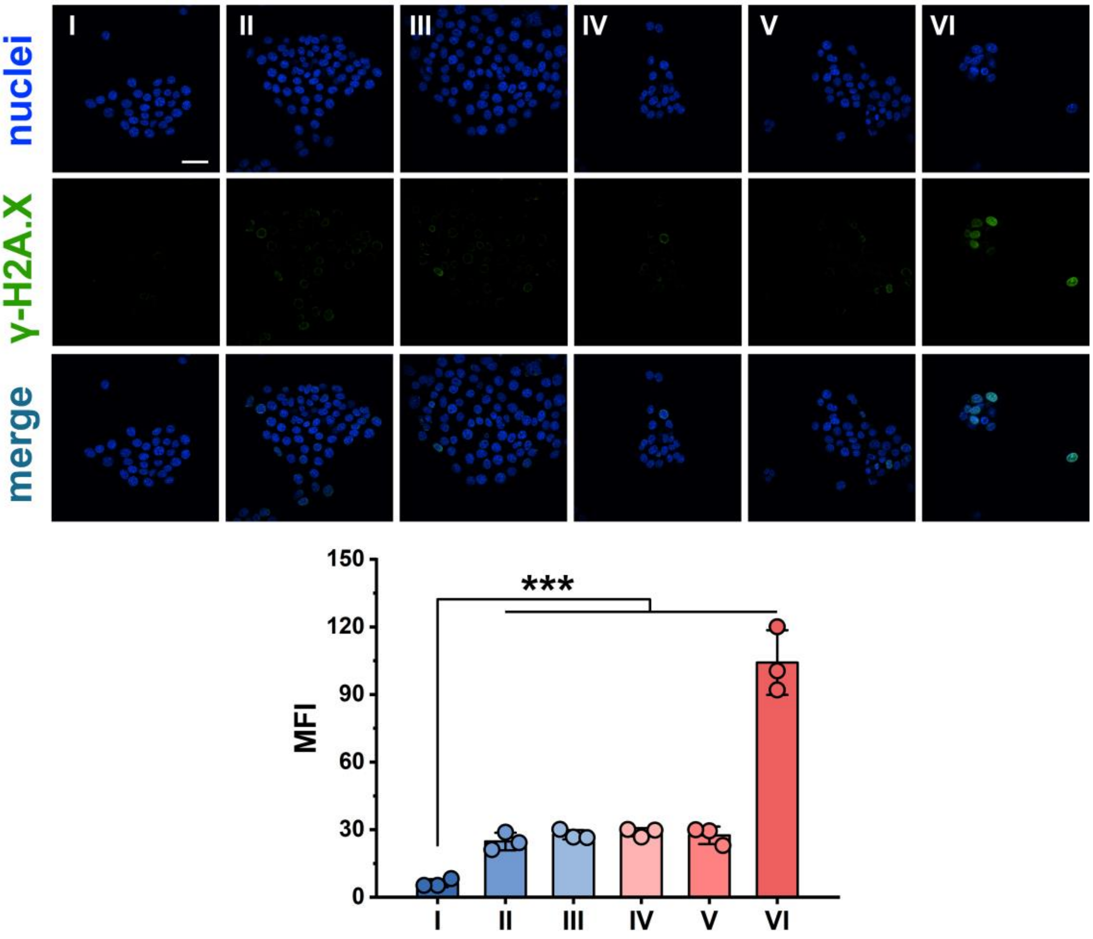

Figure S23. CLSM images and quantitative MFI of JC-1 monomers in 4T1 cells upon different treatments (n = 3). Scale bar is 50 μm. Group: (I) Control, (II) Fe-Gal, (III) Fe-Gal@Fe-cGAMP, (IV) Fe-Gal@Fe-Zol, (V) Fe-Gal@Fe-Zol-cGAMP, (VI) DOX@Fe-Gal@Fe-Zol-cGAMP. Data are shown as mean ± SD; n represents the number of biologically independent samples. *$P < 0.05$, **$P < 0.01$, and ***$P < 0.001$.

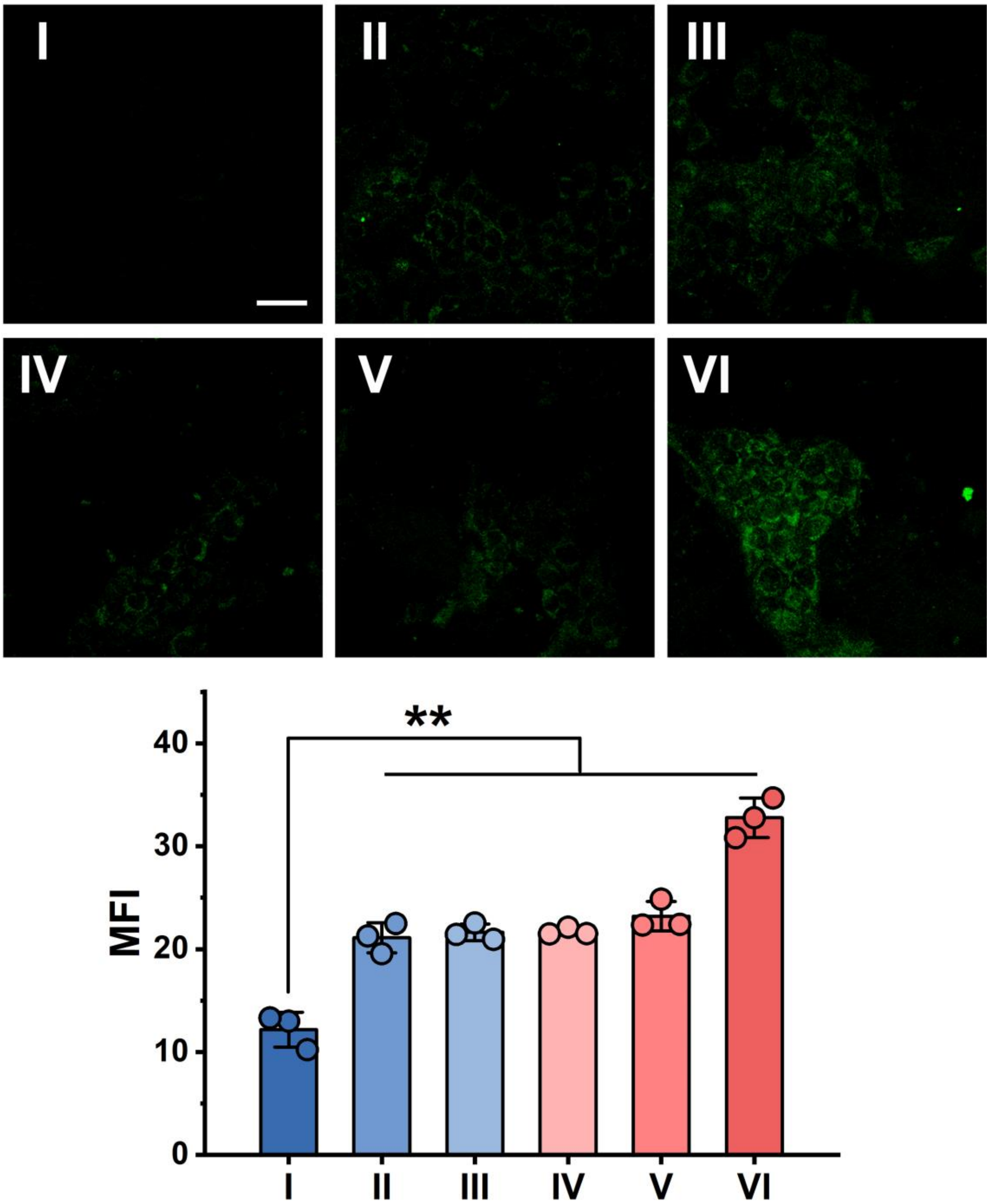

Figure S24. CCK8 assay of 4T1 cells after different treatments (n = 5). Group in (a): (I) Control, (II) DOX@Fe-Gal@Fe-Zol-cGAMP, (III) DOX@Fe-Gal@Fe-Zol-cGAMP + DMSO, (IV) DOX@Fe-Gal@Fe-Zol-cGAMP + 2-Bromohexadecanoic acid. Group in (b): (I) Control, (II) DOX@Fe-Gal@Fe-Zol-cGAMP, (III) DOX@Fe-Gal@Fe-Zol-cGAMP + DMSO, (IV) DOX@Fe-Gal@Fe-Zol-cGAMP + Ac-DEVD-CHO. Data are shown as mean ± SD; n represents the number of biologically independent samples.

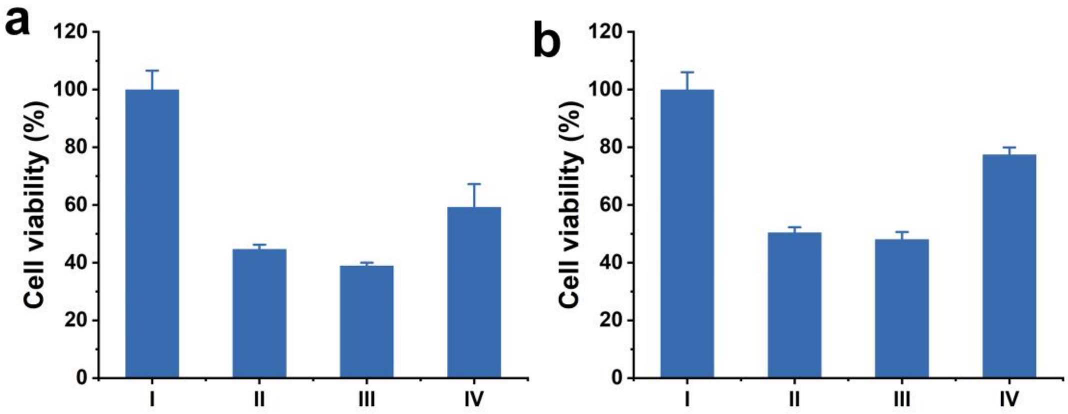

Figure S25. CLSM images of HMGB1 in 4T1 cells upon different treatments. Scale bar is 50 μm. Group: (I) Control, (II) Fe-Gal, (III) Fe-Gal@Fe-cGAMP, (IV) Fe-Gal@Fe-Zol, (V) Fe-Gal@Fe-Zol-cGAMP, (VI) DOX@Fe-Gal@Fe-Zol-cGAMP.

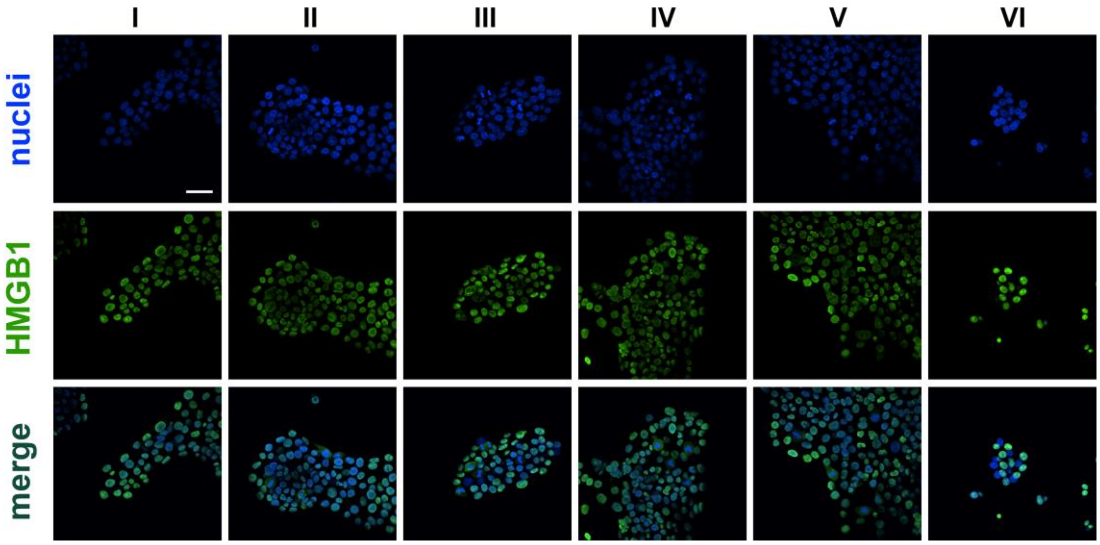

Figure S26. CLSM images of CRT in 4T1 cells upon different treatments. Scale bar is 25 μm. Group: (I) Control, (II) Fe-Gal, (III) Fe-Gal@Fe-cGAMP, (IV) Fe-Gal@Fe-Zol, (V) Fe-Gal@Fe-Zol-cGAMP, (VI) DOX@Fe-Gal@Fe-Zol-cGAMP.

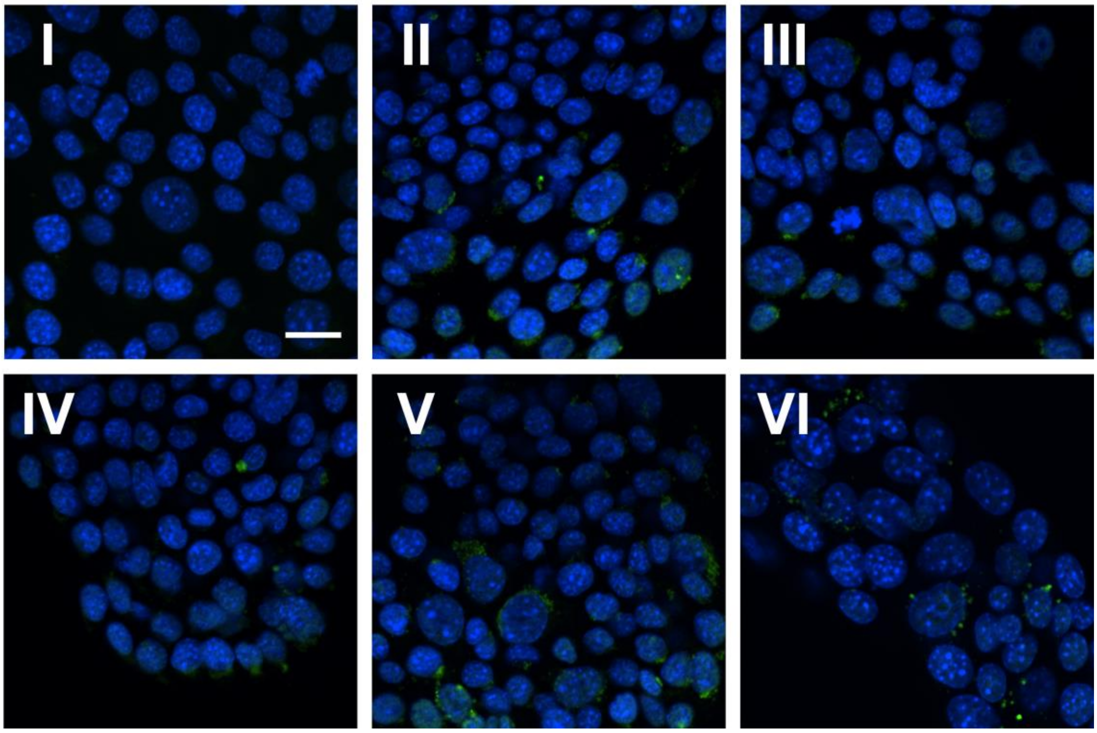

Figure S27. CLSM images of dsDNA via Picogreen staining in 4T1 cells upon different treatments. Scale bar is 25 μm. Group: (I) Control, (II) Fe-Gal, (III) Fe-Gal@Fe-cGAMP, (IV) Fe-Gal@Fe-Zol, (V) Fe-Gal@Fe-Zol-cGAMP, (VI) DOX@Fe-Gal@Fe-Zol-cGAMP.

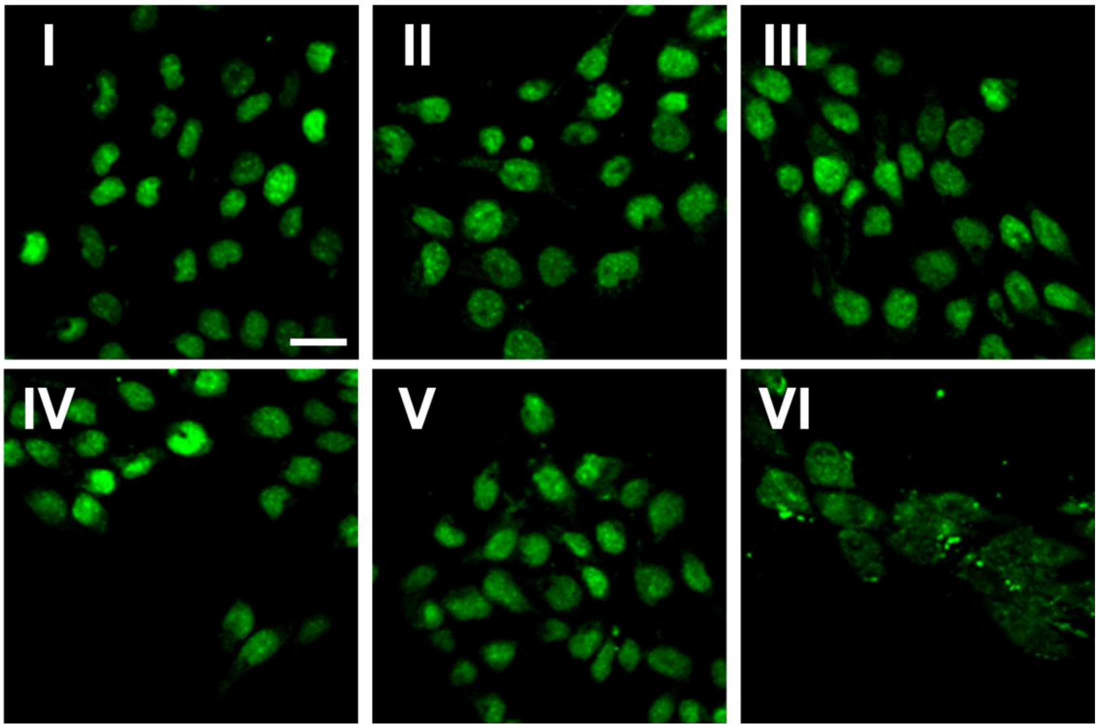

Figure S28. IFN-β secretion levels of 4T1 cells upon different incubation (n = 4). Group: (I) Control, (II) DOX@Fe-Gal@Fe-Zol-cGAMP, (III) DOX@Fe-Gal@Fe-Zol-cGAMP + SN-011, (IV) DOX@Fe-Gal@Fe-Zol-cGAMP + STING-IN-2. Data are shown as mean ± SD; n represents the number of biologically independent samples.

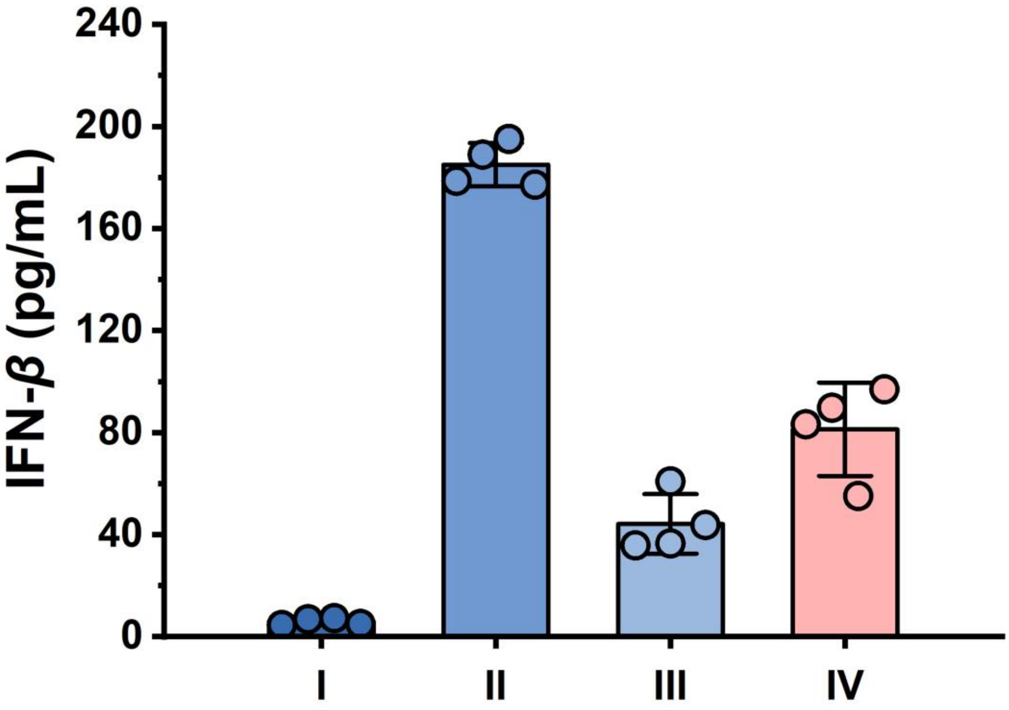

Figure S29. (a) In vivo fluorescence images of orthotopic 4T1 tumor-bearing mice at different time points (0.5, 1, 2.5, 9, 11, 24, and 48 h) after intravenous injection of ICG and ^{ICG}Fe-Gal@Fe-Zol. (b) Ex vivo fluorescence images of major organs and tumors excised from mice at 48 h post-injection of ICG and ^{ICG}Fe-Gal@Fe-Zol.

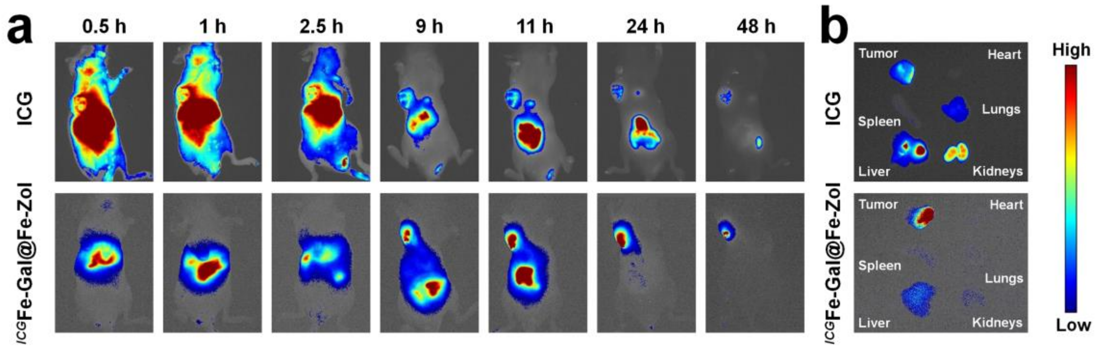

Figure S30. Blood-circulation of intravenously injected ICG and ^{ICG}Fe-Gal@Fe-Zol (n = 3). Data are shown as mean ± SD; n represents the number of biologically independent samples.

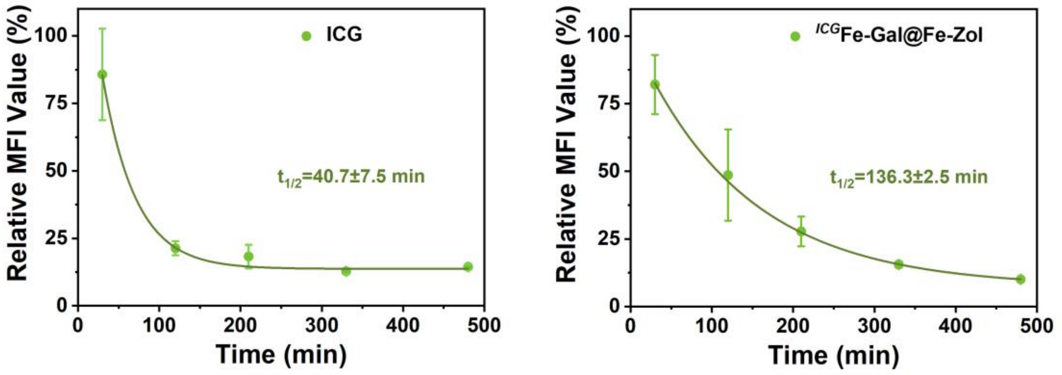

Figure 31. H&E-stained images of tumors collected from mice at the end of various treatments. Scale bar is 100 μm. Group: (I) Control, (II) Fe-Gal, (III) Fe-Gal@Fe-cGAMP, (IV) Fe-Gal@Fe-Zol, (V) Fe-Gal@Fe-Zol-cGAMP, (VI) DOX@Fe-Gal@Fe-Zol-cGAMP.

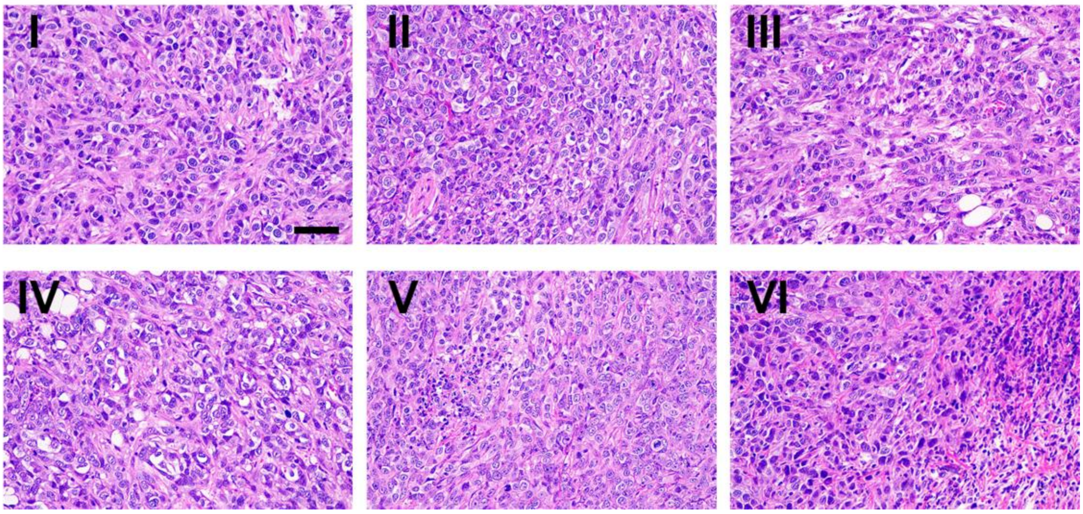

Figure S32. Immunofluorescence images of IFN-*β*, cleaved caspase-3 and GSDME-N in tumors upon different treatments. Scale bar is 100 μm. Group: (I) Control, (II) Fe-Gal, (III) Fe-Gal@Fe-cGAMP, (IV) Fe-Gal@Fe-Zol, (V) Fe-Gal@Fe-Zol-cGAMP, (VI) DOX@Fe-Gal@Fe-Zol-cGAMP.

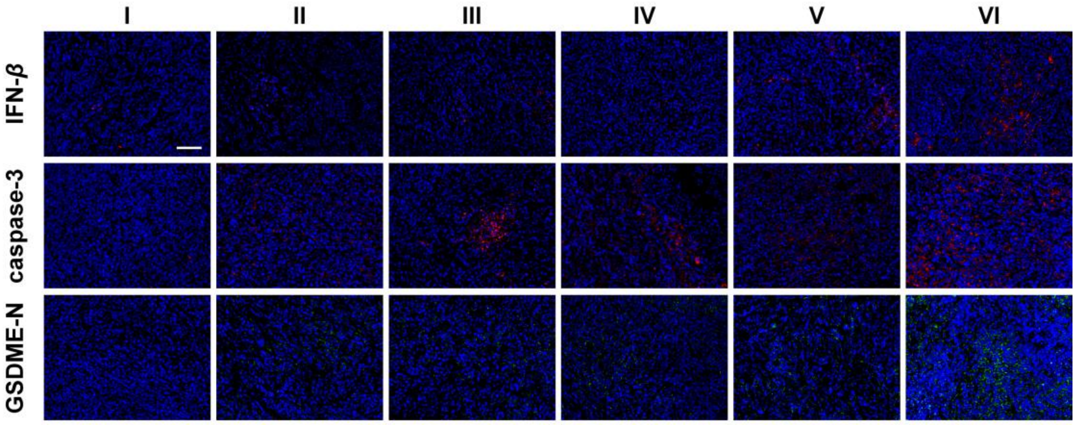

Figure S33. Volumes of tumors (a) and weights of mice (b) in each group throughout treatment period (n = 8). Group: (I) Control, (II) mixture of free drugs, (III) DOX@Fe-Gal@Fe-Zol-cGAMP. Data are shown as mean ± SD; n represents the number of biologically independent samples.

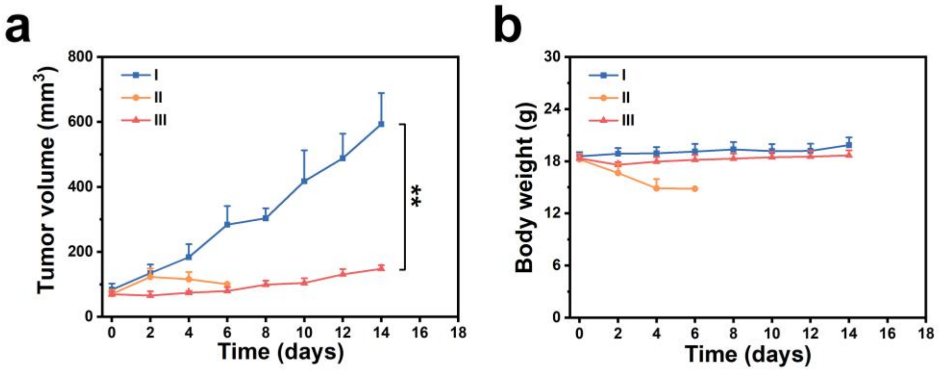

Figure S34. Representative flow cytometric plots of DCs ($CD11c^+CD86^+CD86^+$, gated on $CD11c^+$) in tumors upon different treatments. (I) Control, (II) Fe-Gal, (III) Fe-Gal@Fe-cGAMP, (IV) Fe-Gal@Fe-Zol, (V) Fe-Gal@Fe-Zol-cGAMP, (VI) DOX@Fe-Gal@Fe-Zol-cGAMP.

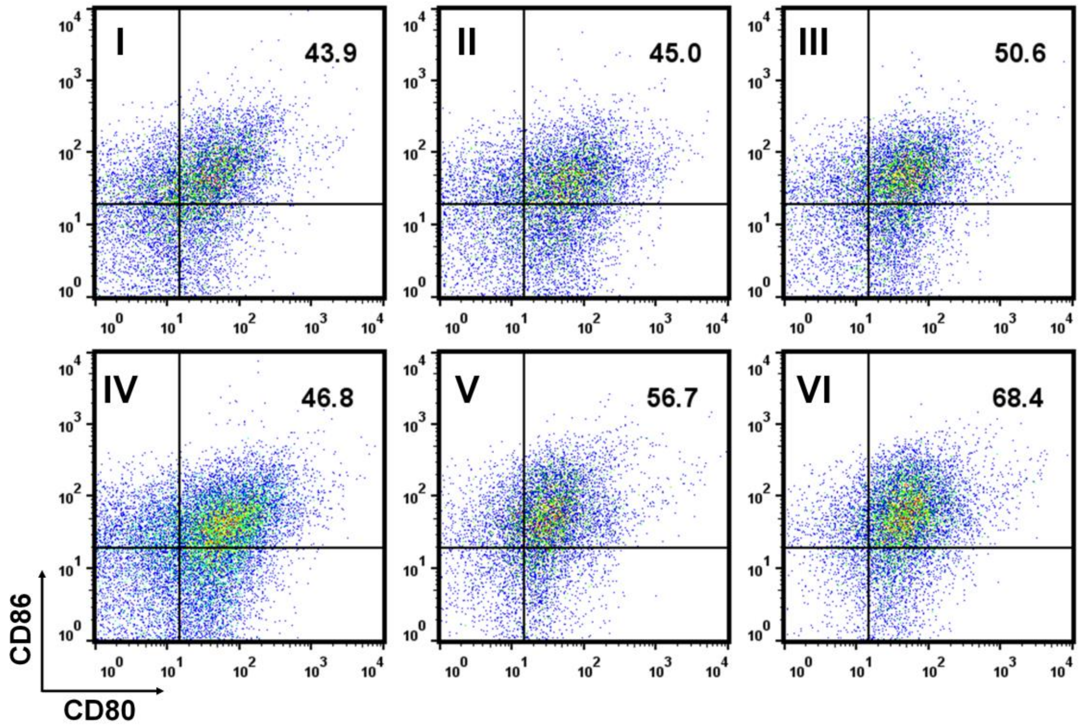

Figure S35. Representative flow cytometric plots of CD3$^+$CD4$^+$ and CD3$^+$CD8$^+$ T cells (gated on CD3$^+$) in tumors upon different treatments. (I) Control, (II) Fe-Gal, (III) Fe-Gal@Fe-cGAMP, (IV) Fe-Gal@Fe-Zol, (V) Fe-Gal@Fe-Zol-cGAMP, (VI) DOX@Fe-Gal@Fe-Zol-cGAMP.

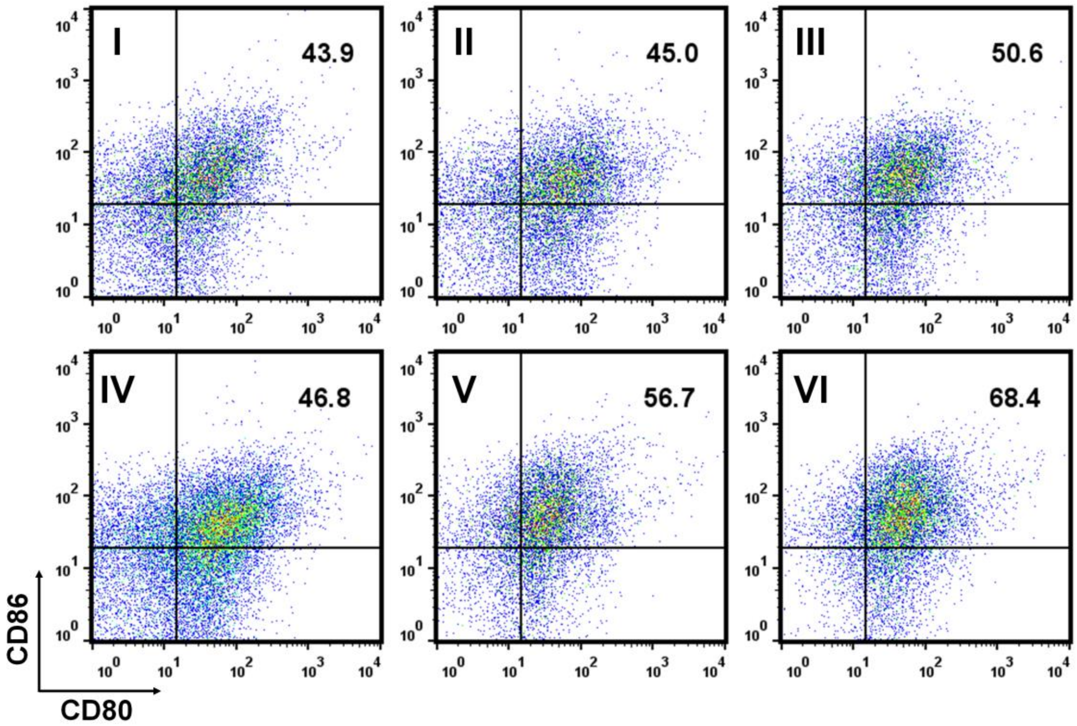

Figure S36. Immunofluorescence images of $CD4^+$ and $CD8^+$ T cells in tumors upon different treatments. Scale bar is 100 μm. Group: (I) Control, (II) Fe-Gal, (III) Fe-Gal@Fe-cGAMP, (IV) Fe-Gal@Fe-Zol, (V) Fe-Gal@Fe-Zol-cGAMP, (VI) DOX@Fe-Gal@Fe-Zol-cGAMP.

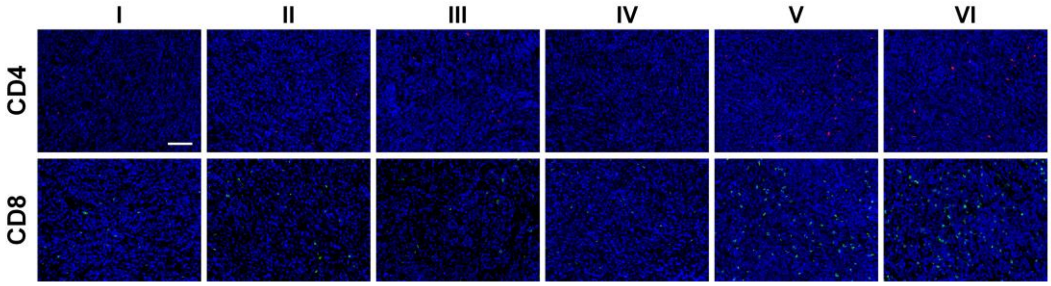

Figure S37. Representative flow cytometric plots of M1-phenotype (F4/80$^+$CD86$^+$, gated on F4/80$^+$) and M2-phenotype (F4/80$^+$CD206$^+$, gated on F4/80$^+$) macrophages in tumors upon different treatments. Group: (I) Control, (II) Fe-Gal, (III) Fe-Gal@Fe-cGAMP, (IV) Fe-Gal@Fe-Zol, (V) Fe-Gal@Fe-Zol-cGAMP, (VI) DOX@Fe-Gal@Fe-Zol-cGAMP.

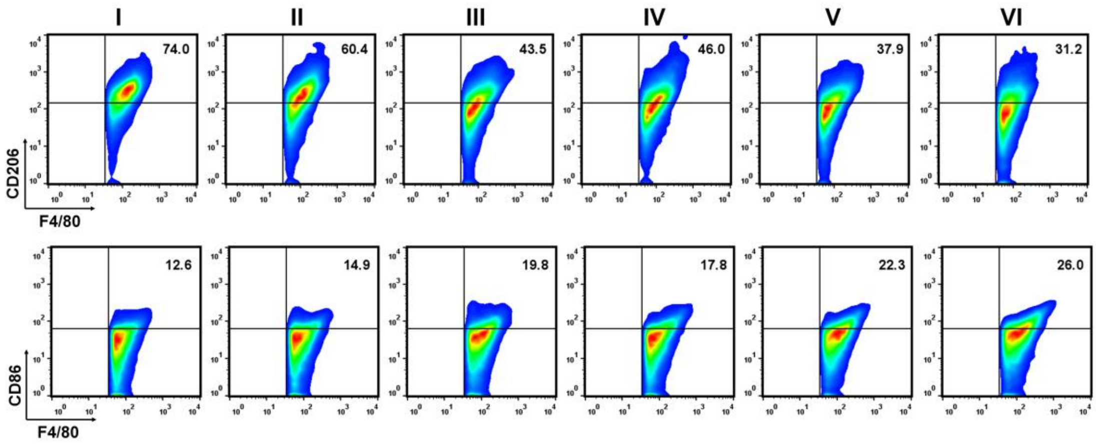

Figure S38. Photograph of lung tissues at 30 days after treatment. Group: (I) Control, (II) Fe-Gal, (III) Fe-Gal@Fe-cGAMP, (IV) Fe-Gal@Fe-Zol, (V) Fe-Gal@Fe-Zol-cGAMP, (VI) DOX@Fe-Gal@Fe-Zol-cGAMP.

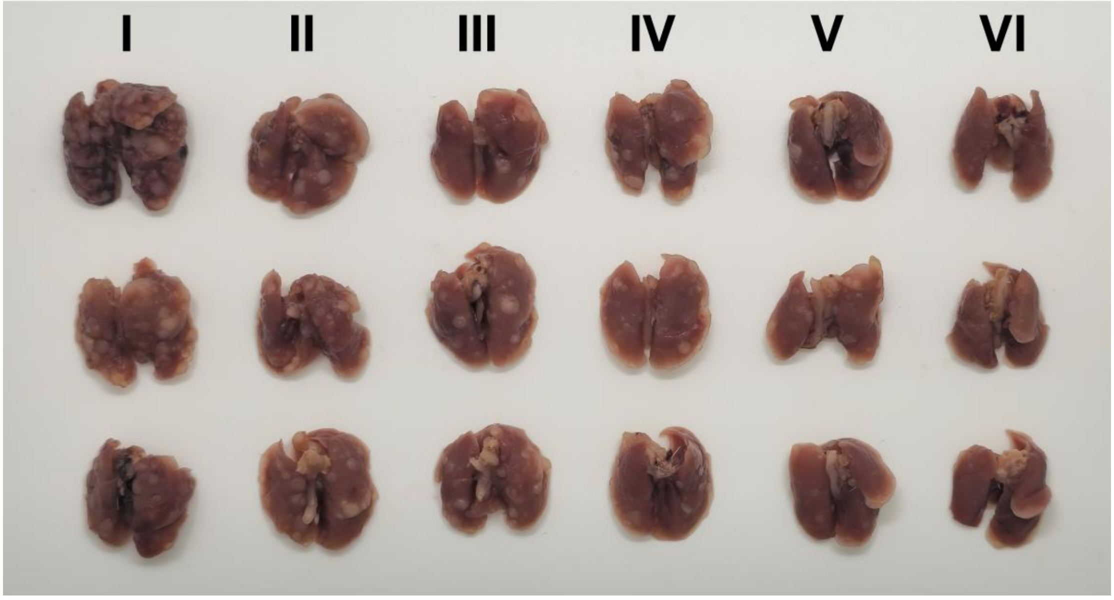

Table.S1. Compositions of DOX@Fe-Gal@Fe-Zol-cGAMP quantified by HPLC and ICP-AES.

**constituent**	**ω (%)**
**galangin**	19.5
**DOX**	7.8
**Fe**	9.7
**cGAMP**	1.2
**zoledronic acid**	61.8
